# Tetra-μ_3_-iodido-tetra­kis­[(tri-*n*-butyl­phosphane-κ*P*)copper(I)]

**DOI:** 10.1107/S1600536814003390

**Published:** 2014-03-05

**Authors:** Simon Klenk, Wolfgang Frey, Martina Bubrin, Sabine Laschat

**Affiliations:** a Institut für Organische Chemie, Universität Stuttgart, Pfaffenwaldring 55, 70569 Stuttgart, Germany; b Institut für Anorganische Chemie, Universität Stuttgart, Pfaffenwaldring 55, 70569 Stuttgart, Germany

## Abstract

The title complex, [Cu_4_I_4_(C_12_H_27_P)_4_], crystallizes with six mol­ecules in the unit cell and with three independent one-third mol­ecule fragments, completed by application of the relevant symmetry operators, in the asymmetric unit. The tetranuclear copper core shows a tetrahedral geometry (site symmetry 3..). The I atoms also form a tetra­hedron, with I⋯I distances of 4.471 (1) Å. Both tetra­hedra show an orientation similar to that of a pair of self-dual platonic bodies. The edges of the I-tetra­hedral structure are capped to the face centers of the Cu-tetra­hedron and *vice versa*. The Cu_face_⋯I distances are 2.18 Å (averaged) and the I_face_⋯Cu distances are 0.78 Å (averaged). As a geometric consequence of these properties there are eight distorted trigonal–bipyramidal polyhedra evident, wherein each trigonal face builds up the equatorial site and the opposite Cu⋯I positions form the axial site. As expected, the *n*-butyl moieties are highly flexible, resulting in large elongations of their anisotropic displacement parameters. Some C atoms of the *n*-butyl groups were needed to fix alternative discrete disordered positions.

## Related literature   

For general background to this work, see: Ainscough *et al.* (2001 ▶); Alyea *et al.* (1985 ▶); Baker *et al.* (1994 ▶); Barron *et al.* (1984 ▶); Bowmaker *et al.* (1989 ▶, 1992 ▶, 1994 ▶ 1999 ▶, 2002 ▶); Churchill & Kalra (1973 ▶, 1974 ▶); Churchill, DeBoer & Donovan (1975 ▶); Churchill, DeBoer & Mendak (1975 ▶); Churchill & Rotella (1977 ▶, 1979 ▶); Dyason, Engelhardt *et al.* (1985 ▶); Dyason, Healy *et al.* (1985 ▶); Gill *et al.* (1976 ▶); Goel & Beauchamp (1983 ▶); Hadjikakou *et al.* (1993 ▶); Herberhold *et al.* (2003 ▶); Hermann *et al.* (2001 ▶); Jansen (1987 ▶); Krause (2002 ▶); Mann *et al.* (1936 ▶); Medina *et al.* (2005 ▶); Moers & Op Het Veld (1970 ▶); Ramaprabhu *et al.* (1993 ▶, 1998 ▶); Schwerdtfeger *et al.* (2004 ▶); Soloveichik *et al.* (1992 ▶); Wells (1936 ▶); Whitesides *et al.* (1971 ▶). The Cu⋯Cu distance is markably short as compared with the reported distances of other tetranuclear copper phosphane complexes (Medina *et al.*, 2005 ▶). Nevertheless there are examples for tetra­meric copper complexes with a Cu⋯Cu distance shorter than 2.700 Å (Blake *et al.*, 2001 ▶; Churchill *et al.*, 1982 ▶; Kim *et al.*, 2008 ▶; Schramm, 1978 ▶). Both tetra­hedra formed by iodines show an orientation similar to that of a pair of self-dual platonic bodies (Glaeser & Polthier, 2010 ▶).
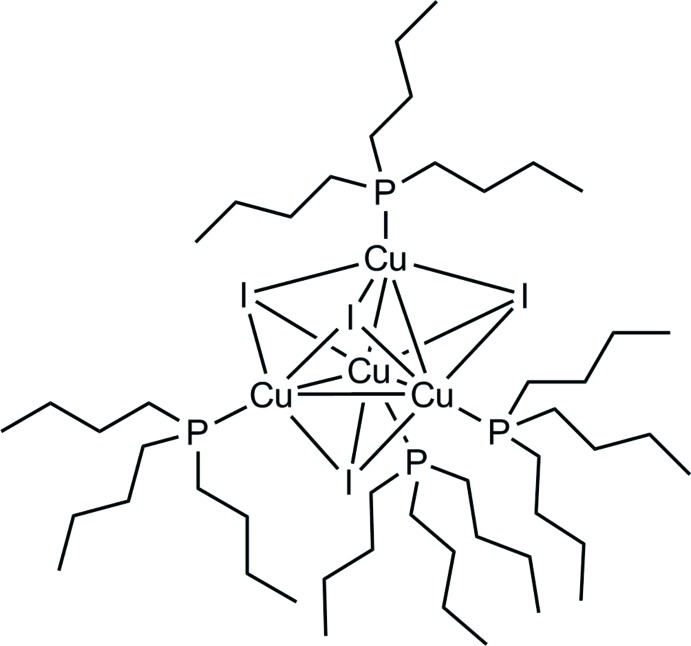


## Experimental   

### 

#### Crystal data  

[Cu_4_I_4_(C_12_H_27_P)_4_]
*M* *_r_* = 1570.98
Trigonal, 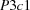

*a* = 22.006 (2) Å
*c* = 23.276 (2) Å
*V* = 9761.6 (15) Å^3^
*Z* = 6
Mo *K*α radiation
μ = 3.31 mm^−1^
*T* = 110 K
0.23 × 0.19 × 0.13 mm

#### Data collection  

Bruker Kappa APEXII DUO diffractometer
Absorption correction: numerical (Blessing, 1995 ▶) *T*_min_ = 0.674, *T*_max_ = 0.852
89010 measured reflections
13415 independent reflections
10102 reflections with *I* > 2σ(*I*)
*R*_int_ = 0.039

#### Refinement  

*R*[*F* ^2^ > 2σ(*F* ^2^)] = 0.046
*wR*(*F* ^2^) = 0.099
*S* = 1.03
13415 reflections
612 parameters
341 restraints
H-atom parameters constrained
Δρ_max_ = 1.62 e Å^−3^
Δρ_min_ = −1.16 e Å^−3^
Absolute structure: Flack (1983 ▶), 6695 Friedel pairs
Absolute structure parameter: −0.02 (2)

### 

Data collection: *APEX2* (Bruker, 2008 ▶); cell refinement: *SAINT* (Bruker, 2008 ▶); data reduction: *SAINT*; program(s) used to solve structure: *SHELXS97* (Sheldrick, 2008 ▶); program(s) used to refine structure: *SHELXL97* (Sheldrick, 2008 ▶); molecular graphics: *XP* in *SHELXTL-Plus* (Sheldrick, 2008 ▶); software used to prepare material for publication: *PLATON* (Spek, 2009 ▶).

## Supplementary Material

Crystal structure: contains datablock(s) I, global. DOI: 10.1107/S1600536814003390/hp2064sup1.cif

Structure factors: contains datablock(s) I. DOI: 10.1107/S1600536814003390/hp2064Isup2.hkl

CCDC reference: 957683

Additional supporting information:  crystallographic information; 3D view; checkCIF report

## Figures and Tables

**Fig. 1.**
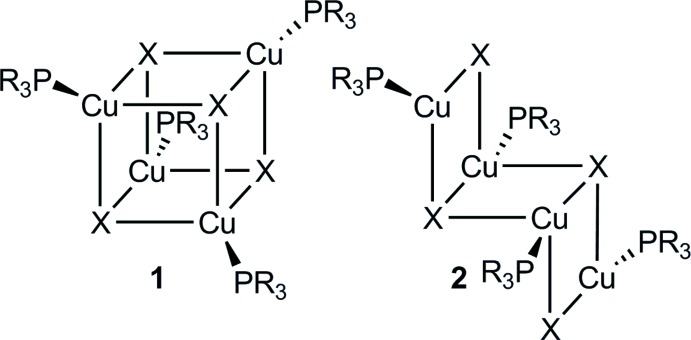
Possible tetrameric core structures based on cubane (1) or open-step framework (2).

**Fig. 2.**
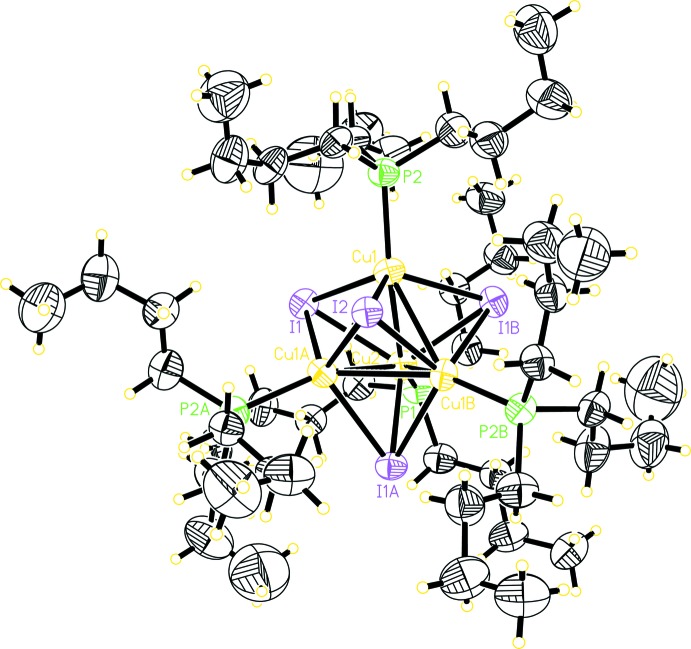
ORTEP-style plot-view of complex [n-Bu3PCuI]4 in the solid state of one conformer.

**Fig. 3.**
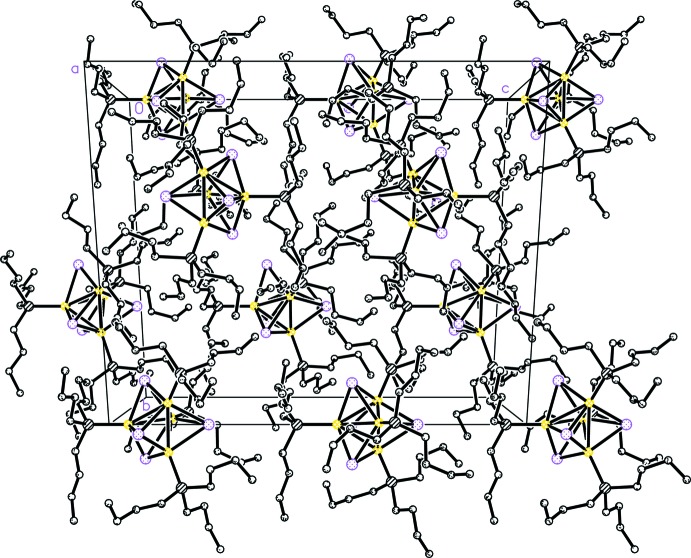
Packing diagram of the unit cell.

**Fig. 4.**
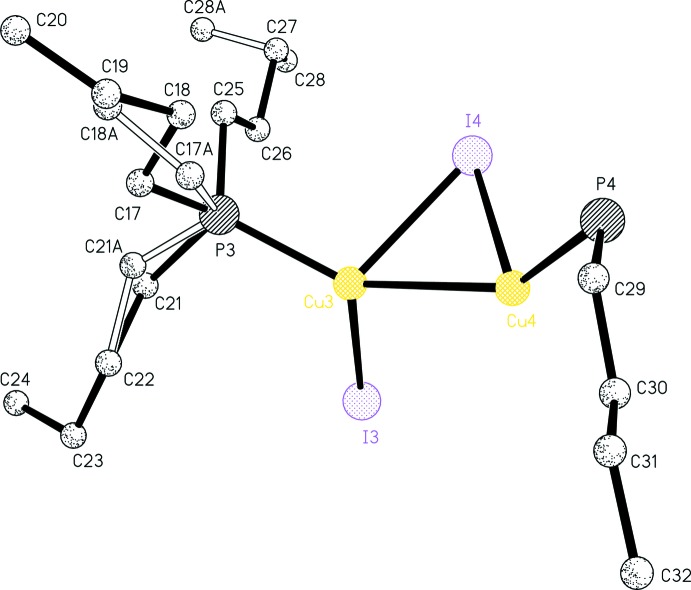
Molecule fragment of independent atom positions of one conformer.

**Fig. 5.**
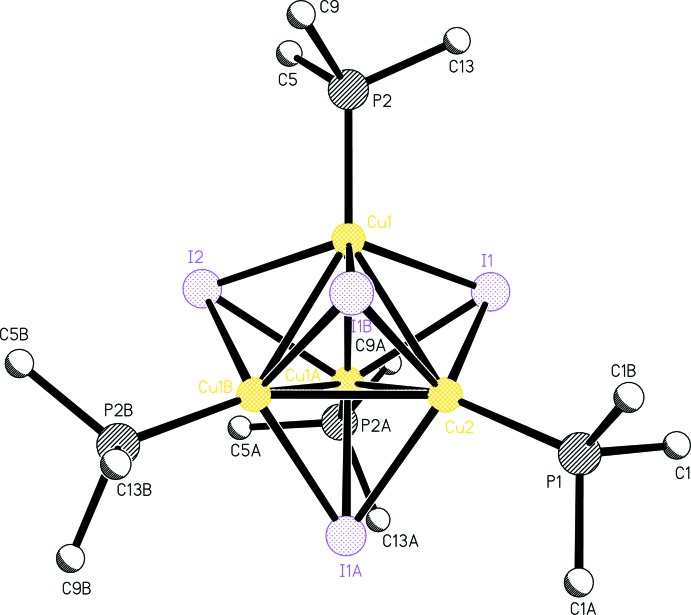
Core geometry.

## supplementary crystallographic information

### 1. Comment

Tetrameric phosphane complexes of copper(I) halides are extensively used as reagents for copper-mediated conjugate additions (Krause, 2002). Furthermore, theoretical interest stems from the fact that all group 11 elements in the oxidation state +1 are prone to form clusters with potential metal-metal distances (Jansen, 1987). Thus, theoretical work on such complexes (XCuP*R*_3_)_4_ has been carried out to study structures and stabilities in detail (Schwerdtfeger *et al.*, 2004; Hermann *et al.*, 2001). However, the plethora of structural information on these compounds came from X-ray crystal structure analyses from various copper(I) halide phosphane complexes (Gill *et al.*, 1976). For monophosphanes, different structural motifs were found, *e.g.* monomers, µ_2_-halide dimers or µ_3_-halide bridged tetramers resulting in coordination numbers of 2, 3, or 4 for copper(I) which seemed to be mostly dependent on the phosphane. Sterically bulky phosphane ligands such as trimesitylphosphane (mes_3_P) (Alyea *et al.*, 1985), tris(2,4,6-trimethoxyphenyl)phosphane (tmpp) (Baker *et al.*, 1994; Bowmaker *et al.*, 1989) or triscycloheptatrienylphosphane (Herberhold *et al.*, 2003) led exclusively to monomeric complexes with a linear *X*–Cu–P orientation. Phosphanes with moderate steric bias resulted in the formation of µ-halide-bridged dimers *e.g.* for PCy_3_, PBu_3_, and mixed aryl phosphanes (Moers & Op Het Veld, 1970; Churchill & Rotella, 1979; Soloveichik *et al.*,1992; Ainscough *et al.*, 2001; Bowmaker *et al.*, 1992; Bowmaker *et al.*, 1994; Hadjikakou *et al.*, 1993; Ramaprabhu *et al.*, 1993; Ramaprabhu *et al.*, 1998). In contrast, sterically less demanding phosphane ligands preferred the formation of tetrameric complexes. In this case, two different structures are possible, a pseudo-cubane structure 1 with triply-bridging halides [Cu(µ_3_-*X*)P*R*_3_]_4_ which was observed for PMe_3_ (*X* = I) (Bowmaker *et al.*, 1999), PEt_3_ (*X* = Cl, Br, I) (Churchill & Kalra, 1974; Churchill, DeBoer, Donovan, 1975; Churchill, DeBoer, Mendak, 1975), *t*-Bu_3_P (*X* = Br, I) (Goel & Beauchamp, 1983; Medina *et al.*, 2005), PMePh_2_ (*X* = I) (Churchill & Rotella, 1977), and PPh_3_ (*X* = Br, Cl, I) or an open-step tetramer 2 which was observed for PPh_3_ (*X* = Br, I) (Churchill & Kalra, 1973; Churchill & Kalra, 1974; Churchill, DeBoer, Donovan, 1975; Churchill, DeBoer, Mendak, 1975; Dyason, Engelhardt *et al.*, 1985; Dyason, Healy *et al.*, 1985) (Fig. 1). It had already been noted that the preferred structure seemed to strongly depend on the solvent, with toluene favoring the cubane structure and chloroform favoring the step isomer while acetonitrile gave mixtures (Dyason, Engelhardt *et al.*, 1985; Dyason, Healy *et al.*, 1985). Similar solvent effects were also reported by Herberhold *et al.* (2003). Although the known tri-*n*-butyl phosphane complex [*n*-Bu_3_PCuI]_4_ had already been characterized by using X-ray crystallography (Wells, 1936), no atomic coordinates, bond lengths or bond angles were reported. Only two space groups *C*3*c* and *C*3*c* were proposed giving preference to the latter (Mann *et al.*, 1936; Wells, 1936). Thus, we decided to reinvestigate the crystal structure of [*n*-Bu_3_PCuI]_4_ (Fig. 2–4). The copper(I) complex was prepared by treatment of anhydrous CuI with *n*-Bu_3_P in a two-phase mixture of a saturated aqueous solution of potassium iodide and diethyl ether (Whitesides *et al.*, 1971). The crude product was dissolved in acetone/methanol (9:1) and cooled to -78°C, whereby the tetrameric complex precipitated. We were able to confirm the previously postulated tetrameric complex with a distorted heterocubane structure similar to the AsEt_3_ derivative (Wells, 1936). Interestingly, [*n*-Bu_3_PCuI]_4_ possesses remarkably short Cu···Cu and large I···I distances with 2.764 (2) Å and 4.471 (1) Å. Further comparison of the structure with other tetrameric copper complexes (Medina *et al.*, 2005) reveals very similar Cu–P bond lengths despite the different σ-donor/π-acceptor strengths of Et_3_P, *n*-Bu_3_P, Ph_3_P and *t*-Bu_3_P, respectively. Furthermore, complex [*n*-Bu_3_PCuI]_4_ has the largest I–Cu–I angle and the smallest Cu–I–Cu angle as compared to the other complexes (Churchill & Kalra, 1974; Dyason, Engelhardt *et al.*, 1985; Dyason, Healy *et al.*, 1985; Medina *et al.*, 2005). Due to this very strong distortion the structure could be better described as two interpenetrating copper and iodine tetrahedrons.

### 2. Experimental

The title complex was prepared analogously to a literature procedure (Whitesides *et al.*, 1971); for characterization, the precipitate obtained at -78°C was dissolved in acetone and crystallized at -50°C. Yield: 46%. *M*.p. 75–76°C [75°C (Mann *et al.*, 1936, Wells, 1936, Whitesides *et al.*, 1971)]. Anal. Calc. for C_48_H_108_Cu_4_I_4_P_4_: C, 36.70; H, 6.93; I, 32.31%. Found: C, 37.09; H, 6.93; I, 32.37%. ^1^H NMR (300 MHz, CDCl_3_): δ = 0.91 (t, J = 7.2 Hz, 9 H, CH_3_), 1.31–1.44 (m, 6 H, CH_2_CH_3_), 1.44–1.64 (m, 12 H, PCH_2_CH_2_) p.p.m.. ^13^C NMR (75 MHz, CDCl_3_): δ = 13.8 (CH_3_), 24.1 (d, J_C—P_ = 16.4 Hz, PCH_2_), 24.6 (d, J_C—P_ = 12.2 Hz, PCH_2_CH_2_), 26.3 (d,J_C—P_ = 2.3 Hz, CH_2_CH_3_) p.p.m.. ^31^P NMR (121 MHz, CDCl_3_): δ = -29.5—34.7 (*m*) p.p.m.. Single crystals suitable for X-ray analysis were obtained by dissolving the product in dichloromethane, overlaying with ethanol and slow evaporation of the solvents at room temperature.

### 3. Refinement

H atoms were only partly located in difference fourier map, because of the strong disordered behaviour of the *n*-butyl moieties. They are refined with fixed individual displacement parameters using a riding model with C—H ranging [U(H) = 1.2 *U*_eq_(C) for methylene groups and [U(H) = 1.5 *U*_eq_(C) for methyl groups] from 0.98 to 0.99 Å. In addition, the methyl groups are allowed to rotate but not to tip. A free refinement of the anisotropic displacement parameters of the *n*-butyl moieties was not possible, so an ISOR = 0.01 instruction for all carbons was established, which solves this problem. The carbon atoms C17, C18, C21, C28, C37 and C38 were identified as discrete disordered atoms. Their distances were fixed by an *DFIX* instruction (intervall 1.50 to 1.54 Å) forced by an estimated standard deviation of 0.01 Å. The population parameters of the disordered positions were refined free. The main domains converged with population fractions of 0.55 (C17, C18), 0.66 (C21), 0.52 (C28) and 0.58 (C37, C38). The distances C22—C23, C23—C24 and C39—C40 were also fixed by the *DFIX* command by the same conditions as above.

Nevertheless it was not possible to prevent the detection of some B-alerts in the checkcif utility. There are two short intermolecular H···H distances of 1.76 Å and 1.95 Å and also a large *U*_eq_(max)/*U*_eq_(min) ratio of the carbon atoms. The C—C bond precision is 0.0205 Å, which is low. All these diagnostic results have their reason in the high flexibility of the *n*-butyl moieties in context to the high electron density localized on the heavy elements (iodine) at the rigid core of the system. Even the terminal carbons show a large elongation of their displacement parameters which is also a sign of the dynamic behaviour of the *n*-butyl moieties. The detection of the large Hirshfeld Test value of bond C23—C24 (7.5 su) yields in the difficulties resolving the discrete disorder positions by the same reasons.

### Crystal data

**Table:**

[Cu_4_I_4_(C_12_H_27_P)_4_]	*D*_x_ = 1.603 Mg m^−^^3^
*M**_r_* = 1570.98	Mo *K*α radiation, λ = 0.71073 Å
Trigonal, *P*3*c*1	Cell parameters from 10102 reflections
Hall symbol: P 3 -2"c	θ = 1.8–26.4°
*a* = 22.006 (2) Å	µ = 3.31 mm^−^^1^
*c* = 23.276 (2) Å	*T* = 110 K
*V* = 9761.6 (15) Å^3^	Irregular, colourless
*Z* = 6	0.23 × 0.19 × 0.13 mm
*F*(000) = 4704	

### Data collection

**Table:**

Bruker Kappa APEXII DUO diffractometer	13415 independent reflections
Radiation source: fine-focus sealed tube	10102 reflections with *I* > 2σ(*I*)
Triumph monochromator	*R*_int_ = 0.039
ω + Phi Scans scans	θ_max_ = 26.4°, θ_min_ = 1.8°
Absorption correction: numerical (Blessing, 1995)	*h* = −27→27
*T*_min_ = 0.674, *T*_max_ = 0.852	*k* = −23→27
89010 measured reflections	*l* = −28→29

### Refinement

**Table:**

Refinement on *F*^2^	Secondary atom site location: difference Fourier map
Least-squares matrix: full	Hydrogen site location: inferred from neighbouring sites
*R*[*F*^2^ > 2σ(*F*^2^)] = 0.046	H-atom parameters constrained
*wR*(*F*^2^) = 0.099	*w* = 1/[σ^2^(*F*_o_^2^) + (0.0213*P*)^2^ + 46.6098*P*] where *P* = (*F*_o_^2^ + 2*F*_c_^2^)/3
*S* = 1.03	(Δ/σ)_max_ = 0.001
13415 reflections	Δρ_max_ = 1.62 e Å^−^^3^
612 parameters	Δρ_min_ = −1.16 e Å^−^^3^
341 restraints	Absolute structure: Flack (1983), 6695 Friedel pairs
Primary atom site location: structure-invariant direct methods	Absolute structure parameter: −0.02 (2)

### Special details

**Table:**

Geometry. All e.s.d.'s (except the e.s.d. in the dihedral angle between two l.s. planes) are estimated using the full covariance matrix. The cell e.s.d.'s are taken into account individually in the estimation of e.s.d.'s in distances, angles and torsion angles; correlations between e.s.d.'s in cell parameters are only used when they are defined by crystal symmetry. An approximate (isotropic) treatment of cell e.s.d.'s is used for estimating e.s.d.'s involving l.s. planes.
Refinement. Refinement of *F*^2^ against ALL reflections. The weighted *R*-factor *wR* and goodness of fit *S* are based on *F*^2^, conventional *R*-factors *R* are based on *F*, with *F* set to zero for negative *F*^2^. The threshold expression of *F*^2^ > σ(*F*^2^) is used only for calculating *R*-factors(gt) *etc*. and is not relevant to the choice of reflections for refinement. *R*-factors based on *F*^2^ are statistically about twice as large as those based on *F*, and *R*- factors based on ALL data will be even larger.

### Fractional atomic coordinates and isotropic or equivalent isotropic displacement parameters (Å^2^)

**Table:**

	*x*	*y*	*z*	*U*_iso_*/*U*_eq_	Occ. (<1)
I1	0.98362 (3)	0.87545 (3)	0.58137 (2)	0.04502 (13)	
I2	1.0000	1.0000	0.73872 (3)	0.0401 (2)	
Cu1	0.92257 (5)	0.93441 (5)	0.64411 (4)	0.0433 (2)	
Cu2	1.0000	1.0000	0.54664 (6)	0.0437 (4)	
P1	1.0000	1.0000	0.45024 (14)	0.0444 (9)	
C1	1.0170 (5)	0.9357 (5)	0.4165 (3)	0.056 (2)	
H1A	0.9796	0.8883	0.4281	0.067*	
H1B	1.0140	0.9394	0.3744	0.067*	
C2	1.0876 (5)	0.9430 (5)	0.4311 (4)	0.055 (2)	
H2A	1.0941	0.9465	0.4733	0.066*	
H2B	1.1254	0.9868	0.4139	0.066*	
C3	1.0940 (6)	0.8809 (6)	0.4090 (4)	0.073 (3)	
H3A	1.0837	0.8748	0.3674	0.087*	
H3B	1.0591	0.8375	0.4288	0.087*	
C4	1.1690 (6)	0.8926 (6)	0.4198 (5)	0.085 (3)	
H4A	1.2032	0.9326	0.3971	0.127*	
H4B	1.1705	0.8504	0.4085	0.127*	
H4C	1.1806	0.9019	0.4607	0.127*	
P2	0.82325 (12)	0.84464 (12)	0.67885 (9)	0.0458 (5)	
C5	0.8325 (5)	0.8140 (5)	0.7491 (3)	0.052 (2)	
H5A	0.8454	0.8521	0.7774	0.063*	
H5B	0.7865	0.7741	0.7608	0.063*	
C6	0.8872 (5)	0.7907 (5)	0.7513 (4)	0.060 (2)	
H6A	0.8666	0.7438	0.7332	0.073*	
H6B	0.9276	0.8235	0.7273	0.073*	
C7	0.9152 (7)	0.7866 (6)	0.8102 (4)	0.084 (3)	
H7A	0.9484	0.7690	0.8054	0.100*	
H7B	0.9417	0.8345	0.8265	0.100*	
C8	0.8628 (8)	0.7430 (8)	0.8497 (6)	0.128 (5)	
H8A	0.8282	0.7584	0.8534	0.192*	
H8B	0.8843	0.7458	0.8872	0.192*	
H8C	0.8395	0.6944	0.8360	0.192*	
C9	0.7468 (5)	0.8566 (6)	0.6880 (4)	0.067 (3)	
H9A	0.7334	0.8661	0.6499	0.081*	
H9B	0.7070	0.8119	0.7022	0.081*	
C10	0.7566 (6)	0.9112 (6)	0.7261 (5)	0.076 (3)	
H10A	0.7946	0.9564	0.7111	0.092*	
H10B	0.7718	0.9030	0.7640	0.092*	
C11	0.6882 (6)	0.9169 (7)	0.7346 (5)	0.091 (4)	
H11A	0.7004	0.9594	0.7575	0.109*	
H11B	0.6716	0.9226	0.6965	0.109*	
C12	0.6287 (7)	0.8531 (8)	0.7646 (6)	0.112 (4)	
H12A	0.6145	0.8111	0.7412	0.168*	
H12B	0.5886	0.8604	0.7697	0.168*	
H12C	0.6448	0.8469	0.8023	0.168*	
C13	0.7867 (5)	0.7657 (5)	0.6361 (3)	0.057 (2)	
H13A	0.8213	0.7496	0.6333	0.068*	
H13B	0.7444	0.7285	0.6555	0.068*	
C14	0.7669 (7)	0.7768 (6)	0.5752 (4)	0.083 (3)	
H14A	0.8080	0.8173	0.5574	0.100*	
H14B	0.7289	0.7883	0.5779	0.100*	
C15	0.7423 (8)	0.7115 (7)	0.5360 (6)	0.108 (4)	
H15A	0.7055	0.6695	0.5562	0.130*	
H15B	0.7214	0.7180	0.5006	0.130*	
C16	0.7988 (13)	0.6998 (12)	0.5209 (9)	0.200 (9)	
H16A	0.8423	0.7450	0.5175	0.300*	
H16B	0.7884	0.6751	0.4840	0.300*	
H16C	0.8042	0.6715	0.5507	0.300*	
I3	0.6667	0.3333	0.63630 (3)	0.0468 (2)	
I4	0.79161 (4)	0.44159 (5)	0.79251 (3)	0.0826 (3)	
Cu3	0.67703 (7)	0.41066 (7)	0.72971 (4)	0.0649 (3)	
Cu4	0.6667	0.3333	0.82528 (8)	0.0834 (7)	
P3	0.69157 (19)	0.51440 (17)	0.70139 (16)	0.0875 (9)	
C17	0.6509 (10)	0.5582 (10)	0.7297 (6)	0.065 (6)	0.55 (2)
H17A	0.6674	0.6028	0.7086	0.078*	0.55 (2)
H17B	0.5996	0.5292	0.7237	0.078*	0.55 (2)
C18	0.6656 (10)	0.5741 (11)	0.7941 (6)	0.070 (6)	0.55 (2)
H18A	0.7157	0.6093	0.7998	0.084*	0.55 (2)
H18B	0.6558	0.5308	0.8147	0.084*	0.55 (2)
C17A	0.6452 (14)	0.5360 (11)	0.7697 (12)	0.075 (8)	0.45 (2)
H17C	0.5938	0.5041	0.7687	0.090*	0.45 (2)
H17D	0.6633	0.5284	0.8064	0.090*	0.45 (2)
C18A	0.6621 (12)	0.6124 (10)	0.7656 (8)	0.075 (8)	0.45 (2)
H18C	0.6428	0.6224	0.7308	0.090*	0.45 (2)
H18D	0.7127	0.6470	0.7700	0.090*	0.45 (2)
C19	0.6189 (8)	0.6031 (8)	0.8202 (6)	0.140 (6)	
H19A	0.5706	0.5712	0.8063	0.168*	0.55 (2)
H19B	0.6181	0.5956	0.8622	0.168*	0.55 (2)
H19C	0.5690	0.5661	0.8168	0.168*	0.45 (2)
H19D	0.6401	0.5969	0.8556	0.168*	0.45 (2)
C20	0.6311 (10)	0.6757 (9)	0.8125 (8)	0.158 (7)	
H20A	0.6784	0.7096	0.8259	0.237*	
H20B	0.5963	0.6811	0.8347	0.237*	
H20C	0.6267	0.6839	0.7717	0.237*	
C21	0.6658 (7)	0.5116 (13)	0.6206 (13)	0.083 (8)	0.66 (5)
H21A	0.6829	0.5598	0.6060	0.100*	0.66 (5)
H21B	0.6868	0.4894	0.5971	0.100*	0.66 (5)
C21A	0.6529 (13)	0.5238 (13)	0.6461 (14)	0.054 (10)	0.34 (5)
H21C	0.6448	0.5630	0.6559	0.065*	0.34 (5)
H21D	0.6886	0.5416	0.6152	0.065*	0.34 (5)
C22	0.5843 (6)	0.4680 (7)	0.6172 (6)	0.118 (5)	
H22A	0.5651	0.4974	0.6314	0.141*	0.66 (5)
H22B	0.5677	0.4273	0.6433	0.141*	0.66 (5)
H22C	0.5493	0.4789	0.6335	0.141*	0.34 (5)
H22D	0.5754	0.4245	0.6369	0.141*	0.34 (5)
C23	0.5541 (11)	0.4406 (10)	0.5560 (6)	0.167 (7)	
H23A	0.5619	0.4013	0.5462	0.201*	
H23B	0.5027	0.4219	0.5568	0.201*	
C24	0.5850 (14)	0.4942 (13)	0.5108 (10)	0.265 (14)	
H24A	0.5850	0.5367	0.5232	0.397*	
H24B	0.5574	0.4763	0.4755	0.397*	
H24C	0.6334	0.5052	0.5035	0.397*	
C25	0.7827 (6)	0.5870 (6)	0.6999 (5)	0.092 (4)	
H25A	0.7832	0.6305	0.6881	0.110*	
H25B	0.8025	0.5944	0.7392	0.110*	
C26	0.8288 (7)	0.5735 (6)	0.6590 (5)	0.095 (4)	
H26A	0.8146	0.5751	0.6189	0.114*	
H26B	0.8214	0.5260	0.6661	0.114*	
C27	0.9069 (8)	0.6276 (8)	0.6663 (7)	0.131 (5)	
H27A	0.9120	0.6747	0.6697	0.157*	0.52 (2)
H27B	0.9235	0.6176	0.7027	0.157*	0.52 (2)
H27C	0.9164	0.6458	0.7061	0.157*	0.48 (2)
H27D	0.9358	0.6056	0.6583	0.157*	0.48 (2)
C28	0.9527 (12)	0.6291 (13)	0.6191 (9)	0.094 (8)	0.52 (2)
H28A	0.9389	0.6421	0.5831	0.141*	0.52 (2)
H28B	0.9479	0.5826	0.6149	0.141*	0.52 (2)
H28C	1.0016	0.6636	0.6279	0.141*	0.52 (2)
C28A	0.9243 (16)	0.6854 (13)	0.6252 (11)	0.112 (11)	0.48 (2)
H28D	0.9752	0.7174	0.6249	0.168*	0.48 (2)
H28E	0.9007	0.7111	0.6369	0.168*	0.48 (2)
H28F	0.9086	0.6660	0.5866	0.168*	0.48 (2)
P4	0.6667	0.3333	0.92200 (18)	0.0867 (17)	
C29	0.5817 (6)	0.3008 (6)	0.9553 (4)	0.083 (3)	
H29A	0.5870	0.2997	0.9974	0.099*	
H29B	0.5647	0.3339	0.9470	0.099*	
C30	0.5275 (6)	0.2290 (7)	0.9353 (5)	0.089 (3)	
H30A	0.5448	0.1960	0.9429	0.107*	
H30B	0.5213	0.2303	0.8933	0.107*	
C31	0.4572 (8)	0.2019 (8)	0.9642 (6)	0.120 (5)	
H31A	0.4392	0.2340	0.9553	0.144*	
H31B	0.4639	0.2028	1.0064	0.144*	
C32	0.4027 (9)	0.1280 (9)	0.9464 (7)	0.146 (6)	
H32A	0.4021	0.1242	0.9045	0.219*	
H32B	0.3563	0.1179	0.9599	0.219*	
H32C	0.4143	0.0944	0.9634	0.219*	
I5	0.3333	0.6667	1.02038 (4)	0.0597 (3)	
I6	0.46756 (4)	0.71941 (4)	0.86390 (2)	0.0751 (2)	
Cu5	0.38394 (7)	0.75018 (7)	0.92661 (4)	0.0679 (3)	
Cu6	0.3333	0.6667	0.83068 (7)	0.0725 (6)	
P5	0.3333	0.6667	0.73393 (16)	0.0726 (14)	
C33	0.3336 (7)	0.5928 (6)	0.7002 (4)	0.084 (3)	
H33A	0.2866	0.5508	0.7054	0.101*	
H33B	0.3410	0.6021	0.6584	0.101*	
C34	0.3888 (6)	0.5754 (6)	0.7225 (4)	0.078 (3)	
H34A	0.4357	0.6180	0.7198	0.093*	
H34B	0.3794	0.5622	0.7636	0.093*	
C35	0.3894 (7)	0.5163 (7)	0.6896 (6)	0.100 (4)	
H35A	0.4016	0.5302	0.6489	0.120*	
H35B	0.3421	0.4741	0.6907	0.120*	
C36	0.4433 (9)	0.4988 (8)	0.7158 (7)	0.137 (6)	
H36A	0.4908	0.5385	0.7099	0.206*	
H36B	0.4390	0.4570	0.6972	0.206*	
H36C	0.4343	0.4900	0.7571	0.206*	
P6	0.44960 (17)	0.86037 (17)	0.95844 (12)	0.0738 (8)	
C37	0.5201 (11)	0.9100 (9)	0.9053 (11)	0.083 (7)	0.58 (2)
H37A	0.5443	0.8827	0.8994	0.100*	0.58 (2)
H37B	0.4963	0.9078	0.8686	0.100*	0.58 (2)
C38	0.5771 (10)	0.9853 (10)	0.9127 (8)	0.104 (9)	0.58 (2)
H38A	0.6072	0.9904	0.9460	0.125*	0.58 (2)
H38B	0.5566	1.0161	0.9184	0.125*	0.58 (2)
C37A	0.5427 (12)	0.9222 (13)	0.9340 (8)	0.053 (7)	0.42 (2)
H37C	0.5649	0.9628	0.9604	0.064*	0.42 (2)
H37D	0.5700	0.8976	0.9357	0.064*	0.42 (2)
C38A	0.5447 (8)	0.9479 (12)	0.8736 (8)	0.065 (8)	0.42 (2)
H38C	0.5133	0.9679	0.8708	0.078*	0.42 (2)
H38D	0.5274	0.9079	0.8465	0.078*	0.42 (2)
C39	0.6193 (8)	1.0038 (8)	0.8566 (7)	0.139 (6)	
H39A	0.6362	0.9711	0.8468	0.167*	0.58 (2)
H39B	0.5950	1.0105	0.8235	0.167*	0.58 (2)
H39C	0.6436	0.9767	0.8511	0.167*	0.42 (2)
H39D	0.6135	1.0179	0.8175	0.167*	0.42 (2)
C40	0.6744 (11)	1.0712 (10)	0.8831 (9)	0.192 (8)	
H40A	0.6562	1.1036	0.8869	0.288*	
H40B	0.7162	1.0924	0.8587	0.288*	
H40C	0.6866	1.0617	0.9212	0.288*	
C41	0.4796 (6)	0.8673 (6)	1.0326 (5)	0.076 (3)	
H41A	0.5023	0.9171	1.0446	0.091*	
H41B	0.4382	0.8403	1.0576	0.091*	
C42	0.5304 (7)	0.8411 (7)	1.0420 (5)	0.092 (4)	
H42A	0.5768	0.8759	1.0263	0.110*	
H42B	0.5138	0.7967	1.0207	0.110*	
C43	0.5390 (7)	0.8284 (8)	1.1070 (5)	0.104 (4)	
H43A	0.4924	0.7960	1.1236	0.125*	
H43B	0.5684	0.8061	1.1104	0.125*	
C44	0.5728 (8)	0.8966 (8)	1.1404 (7)	0.126 (5)	
H44A	0.6206	0.9272	1.1263	0.188*	
H44B	0.5744	0.8868	1.1813	0.188*	
H44C	0.5453	0.9201	1.1354	0.188*	
C45	0.4063 (7)	0.9125 (7)	0.9600 (5)	0.089 (3)	
H45A	0.4410	0.9610	0.9717	0.106*	
H45B	0.3906	0.9148	0.9206	0.106*	
C46	0.3447 (6)	0.8859 (6)	0.9992 (5)	0.080 (3)	
H46A	0.3610	0.8878	1.0392	0.096*	
H46B	0.3119	0.8360	0.9899	0.096*	
C47	0.3053 (7)	0.9252 (7)	0.9964 (6)	0.093 (4)	
H47A	0.2896	0.9242	0.9564	0.112*	
H47B	0.3376	0.9749	1.0068	0.112*	
C48	0.2412 (8)	0.8957 (8)	1.0362 (6)	0.110 (4)	
H48A	0.2140	0.8445	1.0321	0.164*	
H48B	0.2118	0.9158	1.0257	0.164*	
H48C	0.2568	0.9077	1.0761	0.164*	

### Atomic displacement parameters (Å^2^)

**Table:**

	*U*^11^	*U*^22^	*U*^33^	*U*^12^	*U*^13^	*U*^23^
I1	0.0568 (3)	0.0457 (3)	0.0341 (2)	0.0268 (3)	0.0013 (2)	−0.0033 (2)
I2	0.0474 (3)	0.0474 (3)	0.0255 (4)	0.02371 (16)	0.000	0.000
Cu1	0.0470 (6)	0.0423 (5)	0.0341 (5)	0.0175 (5)	0.0006 (4)	−0.0003 (4)
Cu2	0.0528 (6)	0.0528 (6)	0.0253 (7)	0.0264 (3)	0.000	0.000
P1	0.0528 (14)	0.0528 (14)	0.0278 (16)	0.0264 (7)	0.000	0.000
C1	0.072 (5)	0.060 (5)	0.036 (4)	0.033 (4)	0.001 (4)	−0.005 (3)
C2	0.064 (5)	0.061 (5)	0.041 (4)	0.032 (4)	0.005 (4)	−0.003 (4)
C3	0.096 (7)	0.077 (6)	0.061 (5)	0.054 (5)	−0.005 (5)	−0.005 (4)
C4	0.089 (7)	0.100 (7)	0.085 (6)	0.062 (6)	0.001 (5)	−0.004 (5)
P2	0.0462 (12)	0.0488 (13)	0.0381 (10)	0.0205 (11)	0.0013 (9)	0.0029 (9)
C5	0.062 (5)	0.052 (5)	0.042 (4)	0.028 (4)	0.004 (4)	0.010 (3)
C6	0.081 (6)	0.049 (5)	0.049 (4)	0.031 (4)	−0.011 (4)	−0.001 (4)
C7	0.109 (7)	0.083 (6)	0.070 (6)	0.056 (6)	−0.017 (5)	−0.002 (5)
C8	0.133 (9)	0.121 (9)	0.116 (8)	0.054 (7)	−0.019 (7)	0.013 (7)
C9	0.067 (6)	0.084 (6)	0.056 (5)	0.042 (5)	0.005 (4)	0.010 (4)
C10	0.075 (6)	0.072 (6)	0.084 (6)	0.038 (5)	0.014 (5)	0.000 (5)
C11	0.078 (6)	0.114 (8)	0.079 (6)	0.046 (6)	0.008 (5)	0.006 (6)
C12	0.104 (8)	0.132 (9)	0.114 (8)	0.071 (7)	−0.010 (6)	0.005 (7)
C13	0.053 (5)	0.057 (5)	0.046 (4)	0.017 (4)	−0.005 (4)	0.004 (4)
C14	0.096 (7)	0.072 (6)	0.052 (5)	0.019 (5)	−0.020 (5)	−0.004 (4)
C15	0.117 (8)	0.087 (7)	0.094 (7)	0.031 (6)	−0.036 (6)	−0.016 (6)
C16	0.209 (13)	0.190 (12)	0.183 (12)	0.087 (9)	0.004 (9)	−0.038 (9)
I3	0.0580 (3)	0.0580 (3)	0.0245 (4)	0.02900 (17)	0.000	0.000
I4	0.0915 (6)	0.1065 (6)	0.0383 (3)	0.0409 (5)	−0.0148 (3)	−0.0186 (3)
Cu3	0.0853 (9)	0.0761 (8)	0.0342 (5)	0.0410 (7)	−0.0001 (5)	−0.0056 (5)
Cu4	0.1118 (12)	0.1118 (12)	0.0266 (9)	0.0559 (6)	0.000	0.000
P3	0.090 (2)	0.0643 (19)	0.104 (2)	0.0353 (17)	−0.0179 (18)	−0.0260 (17)
C17	0.072 (9)	0.070 (9)	0.060 (8)	0.040 (7)	0.006 (7)	0.000 (7)
C18	0.073 (9)	0.066 (9)	0.057 (8)	0.024 (7)	−0.004 (7)	−0.011 (7)
C17A	0.083 (11)	0.070 (11)	0.079 (11)	0.043 (8)	0.014 (8)	0.004 (8)
C18A	0.078 (11)	0.066 (11)	0.076 (11)	0.033 (8)	0.006 (8)	0.009 (8)
C19	0.122 (9)	0.163 (10)	0.105 (8)	0.049 (7)	0.021 (7)	−0.042 (7)
C20	0.138 (10)	0.200 (11)	0.128 (9)	0.078 (8)	0.019 (7)	0.018 (8)
C21	0.094 (10)	0.075 (10)	0.073 (11)	0.037 (7)	0.005 (7)	0.019 (7)
C21A	0.060 (13)	0.057 (12)	0.042 (12)	0.027 (9)	0.005 (8)	−0.012 (8)
C22	0.109 (8)	0.109 (8)	0.136 (9)	0.055 (7)	−0.019 (7)	0.008 (7)
C23	0.171 (11)	0.176 (11)	0.138 (10)	0.074 (8)	−0.036 (8)	0.026 (8)
C24	0.260 (16)	0.269 (17)	0.266 (17)	0.133 (11)	0.001 (10)	−0.017 (10)
C25	0.096 (7)	0.081 (7)	0.099 (7)	0.046 (6)	0.000 (6)	−0.019 (5)
C26	0.098 (7)	0.077 (7)	0.092 (7)	0.031 (6)	−0.006 (6)	−0.010 (5)
C27	0.121 (9)	0.119 (9)	0.133 (9)	0.045 (7)	0.009 (7)	0.001 (7)
C28	0.094 (11)	0.098 (12)	0.093 (11)	0.051 (8)	0.006 (8)	−0.017 (8)
C28A	0.119 (14)	0.112 (14)	0.106 (14)	0.059 (10)	0.003 (9)	−0.010 (9)
P4	0.116 (3)	0.116 (3)	0.028 (2)	0.0581 (14)	0.000	0.000
C29	0.101 (7)	0.104 (7)	0.032 (4)	0.043 (6)	0.009 (4)	0.011 (4)
C30	0.097 (7)	0.103 (7)	0.058 (5)	0.044 (6)	0.000 (5)	0.019 (5)
C31	0.128 (9)	0.116 (8)	0.094 (7)	0.045 (7)	−0.010 (7)	0.008 (6)
C32	0.155 (10)	0.142 (9)	0.120 (9)	0.059 (7)	0.011 (7)	0.019 (7)
I5	0.0761 (5)	0.0761 (5)	0.0270 (4)	0.0380 (2)	0.000	0.000
I6	0.0866 (5)	0.1004 (5)	0.0381 (3)	0.0465 (4)	0.0095 (3)	−0.0011 (3)
Cu5	0.0833 (9)	0.0823 (9)	0.0354 (5)	0.0393 (8)	0.0014 (6)	−0.0012 (5)
Cu6	0.0944 (10)	0.0944 (10)	0.0285 (9)	0.0472 (5)	0.000	0.000
P5	0.095 (2)	0.095 (2)	0.0269 (18)	0.0477 (11)	0.000	0.000
C33	0.089 (7)	0.099 (7)	0.058 (6)	0.042 (6)	0.005 (5)	−0.005 (5)
C34	0.092 (7)	0.090 (7)	0.059 (5)	0.052 (5)	0.002 (5)	−0.004 (5)
C35	0.092 (7)	0.102 (7)	0.102 (7)	0.046 (6)	0.003 (6)	−0.009 (6)
C36	0.150 (10)	0.131 (9)	0.140 (9)	0.078 (8)	0.012 (7)	−0.006 (7)
P6	0.080 (2)	0.0772 (19)	0.0680 (17)	0.0421 (17)	0.0089 (14)	0.0044 (14)
C37	0.085 (10)	0.073 (10)	0.091 (11)	0.039 (8)	−0.008 (8)	−0.004 (8)
C38	0.102 (12)	0.103 (12)	0.103 (12)	0.048 (9)	−0.009 (8)	−0.003 (8)
C37A	0.046 (10)	0.059 (10)	0.049 (9)	0.022 (7)	0.005 (7)	0.003 (7)
C38A	0.060 (10)	0.075 (11)	0.060 (10)	0.033 (8)	0.010 (7)	0.005 (8)
C39	0.133 (9)	0.143 (9)	0.149 (9)	0.074 (7)	0.027 (7)	0.053 (7)
C40	0.195 (12)	0.209 (12)	0.180 (11)	0.106 (9)	−0.007 (8)	0.041 (9)
C41	0.078 (6)	0.070 (6)	0.080 (6)	0.037 (5)	−0.009 (5)	−0.017 (5)
C42	0.104 (7)	0.077 (6)	0.102 (7)	0.051 (6)	−0.014 (6)	−0.016 (5)
C43	0.097 (7)	0.120 (8)	0.099 (7)	0.058 (6)	−0.011 (6)	−0.013 (6)
C44	0.113 (8)	0.143 (9)	0.134 (9)	0.074 (7)	−0.023 (7)	−0.043 (7)
C45	0.103 (7)	0.092 (7)	0.081 (6)	0.056 (6)	0.013 (5)	0.008 (5)
C46	0.093 (7)	0.084 (6)	0.069 (6)	0.050 (5)	0.011 (5)	0.006 (5)
C47	0.096 (7)	0.095 (7)	0.098 (7)	0.055 (6)	0.002 (6)	0.002 (6)
C48	0.117 (8)	0.123 (8)	0.101 (7)	0.068 (7)	0.006 (6)	0.010 (6)

### Geometric parameters (Å, º)

**Table:**

I1—Cu1^i^	2.6578 (11)	C22—H22C	0.9900
I1—Cu2	2.7032 (7)	C22—H22D	0.9900
I1—Cu1	2.7137 (11)	C23—C24	1.470 (10)
I2—Cu1	2.7161 (12)	C23—H23A	0.9900
I2—Cu1^ii^	2.7161 (11)	C23—H23B	0.9900
I2—Cu1^i^	2.7161 (12)	C24—H24A	0.9800
Cu1—P2	2.240 (2)	C24—H24B	0.9800
Cu1—I1^ii^	2.6578 (11)	C24—H24C	0.9800
Cu1—Cu1^ii^	2.7535 (17)	C25—C26	1.526 (16)
Cu1—Cu1^i^	2.7535 (17)	C25—H25A	0.9900
Cu1—Cu2	2.7702 (15)	C25—H25B	0.9900
Cu2—P1	2.244 (4)	C26—C27	1.534 (18)
Cu2—I1^i^	2.7032 (7)	C26—H26A	0.9900
Cu2—I1^ii^	2.7033 (7)	C26—H26B	0.9900
Cu2—Cu1^ii^	2.7701 (15)	C27—C28	1.480 (10)
Cu2—Cu1^i^	2.7702 (15)	C27—C28A	1.481 (10)
P1—C1	1.812 (9)	C27—H27A	0.9900
P1—C1^i^	1.812 (9)	C27—H27B	0.9900
P1—C1^ii^	1.812 (9)	C27—H27C	0.9900
C1—C2	1.520 (12)	C27—H27D	0.9900
C1—H1A	0.9900	C28—H28A	0.9800
C1—H1B	0.9900	C28—H28B	0.9800
C2—C3	1.530 (13)	C28—H28C	0.9800
C2—H2A	0.9900	C28A—H28D	0.9800
C2—H2B	0.9900	C28A—H28E	0.9800
C3—C4	1.559 (15)	C28A—H28F	0.9800
C3—H3A	0.9900	P4—C29^iv^	1.809 (11)
C3—H3B	0.9900	P4—C29	1.809 (11)
C4—H4A	0.9800	P4—C29^iii^	1.809 (11)
C4—H4B	0.9800	C29—C30	1.500 (16)
C4—H4C	0.9800	C29—H29A	0.9900
P2—C13	1.805 (9)	C29—H29B	0.9900
P2—C5	1.819 (8)	C30—C31	1.509 (18)
P2—C9	1.841 (10)	C30—H30A	0.9900
C5—C6	1.527 (13)	C30—H30B	0.9900
C5—H5A	0.9900	C31—C32	1.52 (2)
C5—H5B	0.9900	C31—H31A	0.9900
C6—C7	1.525 (12)	C31—H31B	0.9900
C6—H6A	0.9900	C32—H32A	0.9800
C6—H6B	0.9900	C32—H32B	0.9800
C7—C8	1.410 (17)	C32—H32C	0.9800
C7—H7A	0.9900	I5—Cu5^v^	2.7081 (13)
C7—H7B	0.9900	I5—Cu5^vi^	2.7081 (13)
C8—H8A	0.9800	I5—Cu5	2.7081 (13)
C8—H8B	0.9800	I6—Cu5^vi^	2.6841 (15)
C8—H8C	0.9800	I6—Cu5	2.6872 (14)
C9—C10	1.421 (13)	I6—Cu6	2.6909 (9)
C9—H9A	0.9900	Cu5—P6	2.239 (4)
C9—H9B	0.9900	Cu5—I6^v^	2.6841 (15)
C10—C11	1.583 (15)	Cu5—Cu6	2.7490 (18)
C10—H10A	0.9900	Cu5—Cu5^vi^	2.777 (2)
C10—H10B	0.9900	Cu5—Cu5^v^	2.777 (2)
C11—C12	1.528 (17)	Cu6—P5	2.252 (4)
C11—H11A	0.9900	Cu6—I6^vi^	2.6910 (9)
C11—H11B	0.9900	Cu6—I6^v^	2.6911 (9)
C12—H12A	0.9800	Cu6—Cu5^v^	2.7491 (18)
C12—H12B	0.9800	Cu6—Cu5^vi^	2.7491 (18)
C12—H12C	0.9800	P5—C33^v^	1.809 (11)
C13—C14	1.536 (12)	P5—C33	1.809 (11)
C13—H13A	0.9900	P5—C33^vi^	1.809 (11)
C13—H13B	0.9900	C33—C34	1.534 (15)
C14—C15	1.554 (16)	C33—H33A	0.9900
C14—H14A	0.9900	C33—H33B	0.9900
C14—H14B	0.9900	C34—C35	1.518 (16)
C15—C16	1.43 (2)	C34—H34A	0.9900
C15—H15A	0.9900	C34—H34B	0.9900
C15—H15B	0.9900	C35—C36	1.543 (19)
C16—H16A	0.9800	C35—H35A	0.9900
C16—H16B	0.9800	C35—H35B	0.9900
C16—H16C	0.9800	C36—H36A	0.9800
I3—Cu3^iii^	2.6994 (13)	C36—H36B	0.9800
I3—Cu3	2.6994 (13)	C36—H36C	0.9800
I3—Cu3^iv^	2.6995 (13)	P6—C45	1.823 (12)
I4—Cu3	2.6909 (15)	P6—C41	1.826 (11)
I4—Cu3^iii^	2.6923 (15)	P6—C37	1.85 (2)
I4—Cu4	2.6956 (11)	P6—C37A	1.89 (2)
Cu3—P3	2.240 (4)	C37—C38	1.507 (10)
Cu3—I4^iv^	2.6924 (15)	C37—H37A	0.9900
Cu3—Cu4	2.7400 (19)	C37—H37B	0.9900
Cu3—Cu3^iii^	2.771 (2)	C38—C39	1.535 (10)
Cu3—Cu3^iv^	2.771 (2)	C38—H38A	0.9900
Cu4—P4	2.251 (5)	C38—H38B	0.9900
Cu4—I4^iii^	2.6955 (10)	C37A—C38A	1.508 (10)
Cu4—I4^iv^	2.6958 (10)	C37A—H37C	0.9900
Cu4—Cu3^iii^	2.7398 (19)	C37A—H37D	0.9900
Cu4—Cu3^iv^	2.7399 (19)	C38A—C39	1.530 (10)
P3—C21A	1.61 (3)	C38A—H38C	0.9900
P3—C17	1.739 (17)	C38A—H38D	0.9900
P3—C25	1.836 (13)	C39—C40	1.502 (10)
P3—C21	1.96 (3)	C39—H39A	0.9900
P3—C17A	2.07 (3)	C39—H39B	0.9900
C17—C18	1.538 (10)	C39—H39C	0.9900
C17—H17A	0.9900	C39—H39D	0.9900
C17—H17B	0.9900	C40—H40A	0.9800
C18—C19	1.575 (10)	C40—H40B	0.9800
C18—H18A	0.9900	C40—H40C	0.9800
C18—H18B	0.9900	C41—C42	1.509 (15)
C17A—C18A	1.534 (10)	C41—H41A	0.9900
C17A—H17C	0.9900	C41—H41B	0.9900
C17A—H17D	0.9900	C42—C43	1.567 (17)
C18A—C19	1.538 (10)	C42—H42A	0.9900
C18A—H18C	0.9900	C42—H42B	0.9900
C18A—H18D	0.9900	C43—C44	1.515 (18)
C19—C20	1.492 (9)	C43—H43A	0.9900
C19—H19A	0.9900	C43—H43B	0.9900
C19—H19B	0.9900	C44—H44A	0.9800
C19—H19C	0.9900	C44—H44B	0.9800
C19—H19D	0.9900	C44—H44C	0.9800
C20—H20A	0.9800	C45—C46	1.489 (15)
C20—H20B	0.9800	C45—H45A	0.9900
C20—H20C	0.9800	C45—H45B	0.9900
C21—C22	1.556 (9)	C46—C47	1.501 (15)
C21—H21A	0.9900	C46—H46A	0.9900
C21—H21B	0.9900	C46—H46B	0.9900
C21A—C22	1.544 (10)	C47—C48	1.533 (17)
C21A—H21C	0.9900	C47—H47A	0.9900
C21A—H21D	0.9900	C47—H47B	0.9900
C22—C23	1.559 (9)	C48—H48A	0.9800
C22—H22A	0.9900	C48—H48B	0.9800
C22—H22B	0.9900	C48—H48C	0.9800
			
Cu1^i^—I1—Cu2	62.22 (3)	C23—C22—H22A	108.5
Cu1^i^—I1—Cu1	61.67 (4)	C21A—C22—H22B	103.0
Cu2—I1—Cu1	61.51 (3)	C21—C22—H22B	108.5
Cu1—I2—Cu1^ii^	60.91 (4)	C23—C22—H22B	108.5
Cu1—I2—Cu1^i^	60.91 (4)	H22A—C22—H22B	107.5
Cu1^ii^—I2—Cu1^i^	60.91 (4)	C21A—C22—H22C	102.1
P2—Cu1—I1^ii^	109.25 (7)	C21—C22—H22C	128.9
P2—Cu1—I1	105.70 (7)	C23—C22—H22C	102.1
I1^ii^—Cu1—I1	112.56 (4)	H22A—C22—H22C	22.3
P2—Cu1—I2	104.61 (6)	H22B—C22—H22C	90.3
I1^ii^—Cu1—I2	112.94 (4)	C21A—C22—H22D	102.1
I1—Cu1—I2	111.18 (4)	C21—C22—H22D	100.6
P2—Cu1—Cu1^ii^	146.32 (8)	C23—C22—H22D	102.1
I1^ii^—Cu1—Cu1^ii^	60.17 (4)	H22A—C22—H22D	122.3
I1—Cu1—Cu1^ii^	107.80 (3)	H22B—C22—H22D	14.8
I2—Cu1—Cu1^ii^	59.544 (18)	H22C—C22—H22D	104.8
P2—Cu1—Cu1^i^	141.32 (8)	C24—C23—C22	114.0 (18)
I1^ii^—Cu1—Cu1^i^	109.43 (3)	C24—C23—H23A	108.7
I1—Cu1—Cu1^i^	58.17 (4)	C22—C23—H23A	108.7
I2—Cu1—Cu1^i^	59.544 (18)	C24—C23—H23B	108.7
Cu1^ii^—Cu1—Cu1^i^	60.0	C22—C23—H23B	108.7
P2—Cu1—Cu2	146.10 (7)	H23A—C23—H23B	107.6
I1^ii^—Cu1—Cu2	59.70 (2)	C23—C24—H24A	109.5
I1—Cu1—Cu2	59.06 (2)	C23—C24—H24B	109.5
I2—Cu1—Cu2	109.15 (4)	H24A—C24—H24B	109.5
Cu1^ii^—Cu1—Cu2	60.20 (2)	C23—C24—H24C	109.5
Cu1^i^—Cu1—Cu2	60.20 (2)	H24A—C24—H24C	109.5
P1—Cu2—I1^i^	107.40 (3)	H24B—C24—H24C	109.5
P1—Cu2—I1	107.40 (3)	C26—C25—P3	112.7 (8)
I1^i^—Cu2—I1	111.47 (3)	C26—C25—H25A	109.1
P1—Cu2—I1^ii^	107.40 (3)	P3—C25—H25A	109.1
I1^i^—Cu2—I1^ii^	111.46 (3)	C26—C25—H25B	109.1
I1—Cu2—I1^ii^	111.46 (3)	P3—C25—H25B	109.1
P1—Cu2—Cu1^ii^	144.98 (3)	H25A—C25—H25B	107.8
I1^i^—Cu2—Cu1^ii^	58.08 (3)	C25—C26—C27	112.1 (11)
I1—Cu2—Cu1^ii^	107.62 (5)	C25—C26—H26A	109.2
I1^ii^—Cu2—Cu1^ii^	59.43 (3)	C27—C26—H26A	109.2
P1—Cu2—Cu1^i^	144.98 (3)	C25—C26—H26B	109.2
I1^i^—Cu2—Cu1^i^	59.43 (3)	C27—C26—H26B	109.2
I1—Cu2—Cu1^i^	58.09 (3)	H26A—C26—H26B	107.9
I1^ii^—Cu2—Cu1^i^	107.62 (5)	C28—C27—C28A	67.7 (15)
Cu1^ii^—Cu2—Cu1^i^	59.60 (4)	C28—C27—C26	114.9 (16)
P1—Cu2—Cu1	144.98 (3)	C28A—C27—C26	107.6 (18)
I1^i^—Cu2—Cu1	107.62 (5)	C28—C27—H27A	108.5
I1—Cu2—Cu1	59.43 (3)	C28A—C27—H27A	46.1
I1^ii^—Cu2—Cu1	58.09 (3)	C26—C27—H27A	108.5
Cu1^ii^—Cu2—Cu1	59.60 (4)	C28—C27—H27B	108.5
Cu1^i^—Cu2—Cu1	59.60 (4)	C28A—C27—H27B	141.0
C1—P1—C1^i^	102.6 (3)	C26—C27—H27B	108.5
C1—P1—C1^ii^	102.6 (3)	H27A—C27—H27B	107.5
C1^i^—P1—C1^ii^	102.6 (3)	C28—C27—H27C	133.1
C1—P1—Cu2	115.7 (3)	C28A—C27—H27C	110.2
C1^i^—P1—Cu2	115.7 (3)	C26—C27—H27C	110.2
C1^ii^—P1—Cu2	115.6 (3)	H27A—C27—H27C	67.0
C2—C1—P1	114.9 (6)	H27B—C27—H27C	42.4
C2—C1—H1A	108.5	C28—C27—H27D	43.5
P1—C1—H1A	108.5	C28A—C27—H27D	110.2
C2—C1—H1B	108.5	C26—C27—H27D	110.2
P1—C1—H1B	108.5	H27A—C27—H27D	139.8
H1A—C1—H1B	107.5	H27B—C27—H27D	69.8
C1—C2—C3	112.2 (8)	H27C—C27—H27D	108.5
C1—C2—H2A	109.2	C27—C28—H28A	109.5
C3—C2—H2A	109.2	C27—C28—H28B	109.5
C1—C2—H2B	109.2	C27—C28—H28C	109.5
C3—C2—H2B	109.2	C27—C28A—H28D	109.5
H2A—C2—H2B	107.9	C27—C28A—H28E	109.5
C2—C3—C4	111.2 (9)	H28D—C28A—H28E	109.5
C2—C3—H3A	109.4	C27—C28A—H28F	109.5
C4—C3—H3A	109.4	H28D—C28A—H28F	109.5
C2—C3—H3B	109.4	H28E—C28A—H28F	109.5
C4—C3—H3B	109.4	C29^iv^—P4—C29	103.0 (4)
H3A—C3—H3B	108.0	C29^iv^—P4—C29^iii^	103.0 (4)
C3—C4—H4A	109.5	C29—P4—C29^iii^	103.0 (4)
C3—C4—H4B	109.5	C29^iv^—P4—Cu4	115.3 (3)
H4A—C4—H4B	109.5	C29—P4—Cu4	115.3 (3)
C3—C4—H4C	109.5	C29^iii^—P4—Cu4	115.3 (3)
H4A—C4—H4C	109.5	C30—C29—P4	113.9 (8)
H4B—C4—H4C	109.5	C30—C29—H29A	108.8
C13—P2—C5	102.6 (4)	P4—C29—H29A	108.8
C13—P2—C9	101.6 (5)	C30—C29—H29B	108.8
C5—P2—C9	103.2 (4)	P4—C29—H29B	108.8
C13—P2—Cu1	114.7 (3)	H29A—C29—H29B	107.7
C5—P2—Cu1	114.3 (3)	C29—C30—C31	113.3 (11)
C9—P2—Cu1	118.3 (4)	C29—C30—H30A	108.9
C6—C5—P2	114.0 (6)	C31—C30—H30A	108.9
C6—C5—H5A	108.8	C29—C30—H30B	108.9
P2—C5—H5A	108.8	C31—C30—H30B	108.9
C6—C5—H5B	108.8	H30A—C30—H30B	107.7
P2—C5—H5B	108.8	C30—C31—C32	114.0 (14)
H5A—C5—H5B	107.6	C30—C31—H31A	108.7
C7—C6—C5	117.4 (8)	C32—C31—H31A	108.7
C7—C6—H6A	108.0	C30—C31—H31B	108.7
C5—C6—H6A	108.0	C32—C31—H31B	108.7
C7—C6—H6B	108.0	H31A—C31—H31B	107.6
C5—C6—H6B	108.0	C31—C32—H32A	109.5
H6A—C6—H6B	107.2	C31—C32—H32B	109.5
C8—C7—C6	114.2 (11)	H32A—C32—H32B	109.5
C8—C7—H7A	108.7	C31—C32—H32C	109.5
C6—C7—H7A	108.7	H32A—C32—H32C	109.5
C8—C7—H7B	108.7	H32B—C32—H32C	109.5
C6—C7—H7B	108.7	Cu5^v^—I5—Cu5^vi^	61.70 (4)
H7A—C7—H7B	107.6	Cu5^v^—I5—Cu5	61.69 (5)
C7—C8—H8A	109.5	Cu5^vi^—I5—Cu5	61.69 (5)
C7—C8—H8B	109.5	Cu5^vi^—I6—Cu5	62.27 (6)
H8A—C8—H8B	109.5	Cu5^vi^—I6—Cu6	61.52 (4)
C7—C8—H8C	109.5	Cu5—I6—Cu6	61.48 (4)
H8A—C8—H8C	109.5	P6—Cu5—I6^v^	106.29 (9)
H8B—C8—H8C	109.5	P6—Cu5—I6	107.47 (9)
C10—C9—P2	115.5 (8)	I6^v^—Cu5—I6	112.44 (5)
C10—C9—H9A	108.4	P6—Cu5—I5	106.98 (8)
P2—C9—H9A	108.4	I6^v^—Cu5—I5	111.68 (5)
C10—C9—H9B	108.4	I6—Cu5—I5	111.58 (5)
P2—C9—H9B	108.4	P6—Cu5—Cu6	145.00 (9)
H9A—C9—H9B	107.5	I6^v^—Cu5—Cu6	59.37 (3)
C9—C10—C11	113.3 (10)	I6—Cu5—Cu6	59.33 (3)
C9—C10—H10A	108.9	I5—Cu5—Cu6	108.02 (5)
C11—C10—H10A	108.9	P6—Cu5—Cu5^vi^	145.35 (11)
C9—C10—H10B	108.9	I6^v^—Cu5—Cu5^vi^	108.36 (5)
C11—C10—H10B	108.9	I6—Cu5—Cu5^vi^	58.81 (5)
H10A—C10—H10B	107.7	I5—Cu5—Cu5^vi^	59.15 (2)
C12—C11—C10	113.4 (11)	Cu6—Cu5—Cu5^vi^	59.66 (3)
C12—C11—H11A	108.9	P6—Cu5—Cu5^v^	144.26 (10)
C10—C11—H11A	108.9	I6^v^—Cu5—Cu5^v^	58.92 (5)
C12—C11—H11B	108.9	I6—Cu5—Cu5^v^	108.26 (5)
C10—C11—H11B	108.9	I5—Cu5—Cu5^v^	59.15 (2)
H11A—C11—H11B	107.7	Cu6—Cu5—Cu5^v^	59.66 (3)
C11—C12—H12A	109.5	Cu5^vi^—Cu5—Cu5^v^	60.0
C11—C12—H12B	109.5	P5—Cu6—I6	106.70 (4)
H12A—C12—H12B	109.5	P5—Cu6—I6^vi^	106.70 (4)
C11—C12—H12C	109.5	I6—Cu6—I6^vi^	112.10 (3)
H12A—C12—H12C	109.5	P5—Cu6—I6^v^	106.70 (4)
H12B—C12—H12C	109.5	I6—Cu6—I6^v^	112.10 (3)
C14—C13—P2	112.4 (7)	I6^vi^—Cu6—I6^v^	112.09 (3)
C14—C13—H13A	109.1	P5—Cu6—Cu5	144.32 (3)
P2—C13—H13A	109.1	I6—Cu6—Cu5	59.19 (4)
C14—C13—H13B	109.1	I6^vi^—Cu6—Cu5	108.98 (6)
P2—C13—H13B	109.1	I6^v^—Cu6—Cu5	59.12 (4)
H13A—C13—H13B	107.9	P5—Cu6—Cu5^v^	144.32 (3)
C13—C14—C15	112.9 (10)	I6—Cu6—Cu5^v^	108.98 (6)
C13—C14—H14A	109.0	I6^vi^—Cu6—Cu5^v^	59.11 (4)
C15—C14—H14A	109.0	I6^v^—Cu6—Cu5^v^	59.19 (4)
C13—C14—H14B	109.0	Cu5—Cu6—Cu5^v^	60.68 (6)
C15—C14—H14B	109.0	P5—Cu6—Cu5^vi^	144.32 (3)
H14A—C14—H14B	107.8	I6—Cu6—Cu5^vi^	59.12 (4)
C16—C15—C14	112.2 (14)	I6^vi^—Cu6—Cu5^vi^	59.19 (4)
C16—C15—H15A	109.2	I6^v^—Cu6—Cu5^vi^	108.98 (6)
C14—C15—H15A	109.2	Cu5—Cu6—Cu5^vi^	60.68 (6)
C16—C15—H15B	109.2	Cu5^v^—Cu6—Cu5^vi^	60.68 (6)
C14—C15—H15B	109.2	C33^v^—P5—C33	102.6 (4)
H15A—C15—H15B	107.9	C33^v^—P5—C33^vi^	102.5 (4)
C15—C16—H16A	109.5	C33—P5—C33^vi^	102.6 (4)
C15—C16—H16B	109.5	C33^v^—P5—Cu6	115.7 (4)
H16A—C16—H16B	109.5	C33—P5—Cu6	115.7 (3)
C15—C16—H16C	109.5	C33^vi^—P5—Cu6	115.7 (3)
H16A—C16—H16C	109.5	C34—C33—P5	115.8 (8)
H16B—C16—H16C	109.5	C34—C33—H33A	108.3
Cu3^iii^—I3—Cu3	61.76 (5)	P5—C33—H33A	108.3
Cu3^iii^—I3—Cu3^iv^	61.76 (5)	C34—C33—H33B	108.3
Cu3—I3—Cu3^iv^	61.76 (5)	P5—C33—H33B	108.3
Cu3—I4—Cu3^iii^	61.96 (5)	H33A—C33—H33B	107.4
Cu3—I4—Cu4	61.15 (4)	C35—C34—C33	113.0 (10)
Cu3^iii^—I4—Cu4	61.13 (5)	C35—C34—H34A	109.0
P3—Cu3—I4	105.29 (10)	C33—C34—H34A	109.0
P3—Cu3—I4^iv^	105.90 (11)	C35—C34—H34B	109.0
I4—Cu3—I4^iv^	112.59 (5)	C33—C34—H34B	109.0
P3—Cu3—I3	109.23 (10)	H34A—C34—H34B	107.8
I4—Cu3—I3	111.73 (5)	C34—C35—C36	110.6 (11)
I4^iv^—Cu3—I3	111.68 (5)	C34—C35—H35A	109.5
P3—Cu3—Cu4	142.84 (10)	C36—C35—H35A	109.5
I4—Cu3—Cu4	59.51 (3)	C34—C35—H35B	109.5
I4^iv^—Cu3—Cu4	59.50 (3)	C36—C35—H35B	109.5
I3—Cu3—Cu4	107.93 (5)	H35A—C35—H35B	108.1
P3—Cu3—Cu3^iii^	145.57 (12)	C35—C36—H36A	109.5
I4—Cu3—Cu3^iii^	59.04 (5)	C35—C36—H36B	109.5
I4^iv^—Cu3—Cu3^iii^	108.47 (5)	H36A—C36—H36B	109.5
I3—Cu3—Cu3^iii^	59.12 (2)	C35—C36—H36C	109.5
Cu4—Cu3—Cu3^iii^	59.62 (3)	H36A—C36—H36C	109.5
P3—Cu3—Cu3^iv^	146.15 (11)	H36B—C36—H36C	109.5
I4—Cu3—Cu3^iv^	108.51 (5)	C45—P6—C41	102.1 (5)
I4^iv^—Cu3—Cu3^iv^	58.99 (5)	C45—P6—C37	101.0 (8)
I3—Cu3—Cu3^iv^	59.12 (2)	C41—P6—C37	114.8 (8)
Cu4—Cu3—Cu3^iv^	59.62 (3)	C45—P6—C37A	105.8 (8)
Cu3^iii^—Cu3—Cu3^iv^	60.0	C41—P6—C37A	90.5 (7)
P4—Cu4—I4^iii^	106.43 (4)	C37—P6—C37A	24.4 (7)
P4—Cu4—I4	106.43 (4)	C45—P6—Cu5	115.5 (5)
I4^iii^—Cu4—I4	112.33 (4)	C41—P6—Cu5	114.4 (4)
P4—Cu4—I4^iv^	106.43 (4)	C37—P6—Cu5	108.3 (6)
I4^iii^—Cu4—I4^iv^	112.33 (4)	C37A—P6—Cu5	124.0 (8)
I4—Cu4—I4^iv^	112.33 (4)	C38—C37—P6	124.2 (15)
P4—Cu4—Cu3^iii^	144.28 (3)	C38—C37—H37A	106.3
I4^iii^—Cu4—Cu3^iii^	59.34 (4)	P6—C37—H37A	106.3
I4—Cu4—Cu3^iii^	59.37 (4)	C38—C37—H37B	106.3
I4^iv^—Cu4—Cu3^iii^	109.29 (7)	P6—C37—H37B	106.3
P4—Cu4—Cu3^iv^	144.27 (3)	H37A—C37—H37B	106.4
I4^iii^—Cu4—Cu3^iv^	59.37 (4)	C37—C38—C39	104.7 (14)
I4—Cu4—Cu3^iv^	109.29 (7)	C37—C38—H38A	110.8
I4^iv^—Cu4—Cu3^iv^	59.34 (4)	C39—C38—H38A	110.8
Cu3^iii^—Cu4—Cu3^iv^	60.75 (6)	C37—C38—H38B	110.8
P4—Cu4—Cu3	144.27 (3)	C39—C38—H38B	110.8
I4^iii^—Cu4—Cu3	109.29 (7)	H38A—C38—H38B	108.9
I4—Cu4—Cu3	59.34 (4)	C38A—C37A—P6	111.7 (14)
I4^iv^—Cu4—Cu3	59.38 (4)	C38A—C37A—H37C	109.3
Cu3^iii^—Cu4—Cu3	60.75 (6)	P6—C37A—H37C	109.3
Cu3^iv^—Cu4—Cu3	60.75 (6)	C38A—C37A—H37D	109.3
C21A—P3—C17	77.3 (15)	P6—C37A—H37D	109.3
C21A—P3—C25	106.9 (10)	H37C—C37A—H37D	107.9
C17—P3—C25	99.5 (8)	C37A—C38A—C39	111.2 (15)
C21A—P3—C21	22.1 (11)	C37A—C38A—H38C	109.4
C17—P3—C21	99.2 (11)	C39—C38A—H38C	109.4
C25—P3—C21	100.5 (6)	C37A—C38A—H38D	109.4
C21A—P3—C17A	103.4 (17)	C39—C38A—H38D	109.4
C17—P3—C17A	29.8 (7)	H38C—C38A—H38D	108.0
C25—P3—C17A	105.3 (8)	C40—C39—C38A	134.3 (18)
C21—P3—C17A	125.3 (12)	C40—C39—C38	89.6 (15)
C21A—P3—Cu3	123.4 (9)	C38A—C39—C38	45.9 (11)
C17—P3—Cu3	127.0 (7)	C40—C39—H39A	113.7
C25—P3—Cu3	115.5 (4)	C38A—C39—H39A	96.8
C21—P3—Cu3	111.1 (9)	C38—C39—H39A	113.7
C17A—P3—Cu3	99.9 (6)	C40—C39—H39B	113.7
C18—C17—P3	112.9 (12)	C38A—C39—H39B	83.1
C18—C17—H17A	109.0	C38—C39—H39B	113.7
P3—C17—H17A	109.0	H39A—C39—H39B	111.0
C18—C17—H17B	109.0	C40—C39—H39C	103.6
P3—C17—H17B	109.0	C38A—C39—H39C	103.6
H17A—C17—H17B	107.8	C38—C39—H39C	112.9
C17—C18—C19	111.2 (12)	H39A—C39—H39C	10.3
C17—C18—H18A	109.4	H39B—C39—H39C	119.0
C19—C18—H18A	109.4	C40—C39—H39D	103.6
C17—C18—H18B	109.4	C38A—C39—H39D	103.6
C19—C18—H18B	109.4	C38—C39—H39D	135.2
H18A—C18—H18B	108.0	H39A—C39—H39D	99.9
C18A—C17A—P3	109.1 (14)	H39B—C39—H39D	22.2
C18A—C17A—H17C	109.9	H39C—C39—H39D	105.3
P3—C17A—H17C	109.9	C39—C40—H40A	109.5
C18A—C17A—H17D	109.9	C39—C40—H40B	109.5
P3—C17A—H17D	109.9	H40A—C40—H40B	109.5
H17C—C17A—H17D	108.3	C39—C40—H40C	109.5
C17A—C18A—C19	90.4 (14)	H40A—C40—H40C	109.5
C17A—C18A—H18C	113.6	H40B—C40—H40C	109.5
C19—C18A—H18C	113.6	C42—C41—P6	114.3 (8)
C17A—C18A—H18D	113.6	C42—C41—H41A	108.7
C19—C18A—H18D	113.6	P6—C41—H41A	108.7
H18C—C18A—H18D	110.8	C42—C41—H41B	108.7
C20—C19—C18A	89.4 (13)	P6—C41—H41B	108.7
C20—C19—C18	125.6 (15)	H41A—C41—H41B	107.6
C18A—C19—C18	41.6 (10)	C41—C42—C43	112.8 (11)
C20—C19—H19A	105.9	C41—C42—H42A	109.0
C18A—C19—H19A	101.5	C43—C42—H42A	109.0
C18—C19—H19A	105.9	C41—C42—H42B	109.0
C20—C19—H19B	105.9	C43—C42—H42B	109.0
C18A—C19—H19B	142.8	H42A—C42—H42B	107.8
C18—C19—H19B	105.9	C44—C43—C42	111.3 (12)
H19A—C19—H19B	106.3	C44—C43—H43A	109.4
C20—C19—H19C	113.7	C42—C43—H43A	109.4
C18A—C19—H19C	113.7	C44—C43—H43B	109.4
C18—C19—H19C	108.5	C42—C43—H43B	109.4
H19A—C19—H19C	15.3	H43A—C43—H43B	108.0
H19B—C19—H19C	91.2	C43—C44—H44A	109.5
C20—C19—H19D	113.7	C43—C44—H44B	109.5
C18A—C19—H19D	113.7	H44A—C44—H44B	109.5
C18—C19—H19D	79.4	C43—C44—H44C	109.5
H19A—C19—H19D	126.0	H44A—C44—H44C	109.5
H19B—C19—H19D	29.1	H44B—C44—H44C	109.5
H19C—C19—H19D	111.0	C46—C45—P6	115.1 (8)
C19—C20—H20A	109.5	C46—C45—H45A	108.5
C19—C20—H20B	109.5	P6—C45—H45A	108.5
H20A—C20—H20B	109.5	C46—C45—H45B	108.5
C19—C20—H20C	109.5	P6—C45—H45B	108.5
H20A—C20—H20C	109.5	H45A—C45—H45B	107.5
H20B—C20—H20C	109.5	C45—C46—C47	114.9 (10)
C22—C21—P3	107.2 (14)	C45—C46—H46A	108.5
C22—C21—H21A	110.3	C47—C46—H46A	108.5
P3—C21—H21A	110.3	C45—C46—H46B	108.5
C22—C21—H21B	110.3	C47—C46—H46B	108.5
P3—C21—H21B	110.3	H46A—C46—H46B	107.5
H21A—C21—H21B	108.5	C46—C47—C48	113.8 (11)
C22—C21A—P3	127.9 (19)	C46—C47—H47A	108.8
C22—C21A—H21C	105.3	C48—C47—H47A	108.8
P3—C21A—H21C	105.3	C46—C47—H47B	108.8
C22—C21A—H21D	105.3	C48—C47—H47B	108.8
P3—C21A—H21D	105.3	H47A—C47—H47B	107.7
H21C—C21A—H21D	106.0	C47—C48—H48A	109.5
C21A—C22—C21	28.5 (10)	C47—C48—H48B	109.5
C21A—C22—C23	139.8 (18)	H48A—C48—H48B	109.5
C21—C22—C23	115.0 (17)	C47—C48—H48C	109.5
C21A—C22—H22A	84.4	H48A—C48—H48C	109.5
C21—C22—H22A	108.5	H48B—C48—H48C	109.5
			
Cu1^i^—I1—Cu1—P2	−141.25 (7)	I4—Cu3—P3—C17A	−89.2 (9)
Cu2—I1—Cu1—P2	146.80 (7)	I4^iv^—Cu3—P3—C17A	30.3 (9)
Cu1^i^—I1—Cu1—I1^ii^	99.56 (4)	I3—Cu3—P3—C17A	150.7 (9)
Cu2—I1—Cu1—I1^ii^	27.61 (3)	Cu4—Cu3—P3—C17A	−29.8 (9)
Cu1^i^—I1—Cu1—I2	−28.30 (4)	Cu3^iii^—Cu3—P3—C17A	−146.2 (9)
Cu2—I1—Cu1—I2	−100.25 (4)	Cu3^iv^—Cu3—P3—C17A	87.8 (9)
Cu1^i^—I1—Cu1—Cu1^ii^	35.178 (18)	C21A—P3—C17—C18	−178.8 (18)
Cu2—I1—Cu1—Cu1^ii^	−36.77 (3)	C25—P3—C17—C18	−73.5 (16)
Cu2—I1—Cu1—Cu1^i^	−71.95 (3)	C21—P3—C17—C18	−175.9 (15)
Cu1^i^—I1—Cu1—Cu2	71.95 (3)	C17A—P3—C17—C18	30.7 (17)
Cu1^ii^—I2—Cu1—P2	−147.60 (9)	Cu3—P3—C17—C18	58.8 (17)
Cu1^i^—I2—Cu1—P2	141.49 (9)	P3—C17—C18—C19	−171.3 (13)
Cu1^ii^—I2—Cu1—I1^ii^	−28.89 (4)	C21A—P3—C17A—C18A	−63 (2)
Cu1^i^—I2—Cu1—I1^ii^	−99.80 (3)	C17—P3—C17A—C18A	−33.8 (17)
Cu1^ii^—I2—Cu1—I1	98.76 (3)	C25—P3—C17A—C18A	49 (2)
Cu1^i^—I2—Cu1—I1	27.86 (4)	C21—P3—C17A—C18A	−67 (2)
Cu1^i^—I2—Cu1—Cu1^ii^	−70.907 (16)	Cu3—P3—C17A—C18A	168.6 (18)
Cu1^ii^—I2—Cu1—Cu1^i^	70.907 (16)	P3—C17A—C18A—C19	179.1 (15)
Cu1^ii^—I2—Cu1—Cu2	35.453 (8)	C17A—C18A—C19—C20	−175.6 (19)
Cu1^i^—I2—Cu1—Cu2	−35.454 (8)	C17A—C18A—C19—C18	31.7 (14)
Cu1^i^—I1—Cu2—P1	144.67 (3)	C17—C18—C19—C20	−76 (2)
Cu1—I1—Cu2—P1	−144.26 (3)	C17—C18—C19—C18A	−42.1 (17)
Cu1^i^—I1—Cu2—I1^i^	27.29 (4)	C21A—P3—C21—C22	−55 (2)
Cu1—I1—Cu2—I1^i^	98.36 (5)	C17—P3—C21—C22	−62.2 (18)
Cu1^i^—I1—Cu2—I1^ii^	−97.95 (5)	C25—P3—C21—C22	−163.8 (15)
Cu1—I1—Cu2—I1^ii^	−26.88 (4)	C17A—P3—C21—C22	−46 (2)
Cu1^i^—I1—Cu2—Cu1^ii^	−34.60 (4)	Cu3—P3—C21—C22	73.5 (18)
Cu1—I1—Cu2—Cu1^ii^	36.47 (4)	C17—P3—C21A—C22	−103 (3)
Cu1—I1—Cu2—Cu1^i^	71.07 (5)	C25—P3—C21A—C22	161 (3)
Cu1^i^—I1—Cu2—Cu1	−71.07 (5)	C21—P3—C21A—C22	85 (3)
P2—Cu1—Cu2—P1	5.30 (15)	C17A—P3—C21A—C22	−88 (3)
I1^ii^—Cu1—Cu2—P1	−74.04 (4)	Cu3—P3—C21A—C22	23 (4)
I1—Cu1—Cu2—P1	76.24 (4)	P3—C21A—C22—C21	−99 (4)
I2—Cu1—Cu2—P1	180.0	P3—C21A—C22—C23	−135 (3)
Cu1^ii^—Cu1—Cu2—P1	−144.816 (9)	P3—C21—C22—C21A	42 (3)
Cu1^i^—Cu1—Cu2—P1	144.815 (9)	P3—C21—C22—C23	−162.5 (15)
P2—Cu1—Cu2—I1^i^	−175.92 (13)	C21A—C22—C23—C24	−30 (4)
I1^ii^—Cu1—Cu2—I1^i^	104.743 (17)	C21—C22—C23—C24	−48 (3)
I1—Cu1—Cu2—I1^i^	−104.973 (17)	C21A—P3—C25—C26	−80.9 (17)
I2—Cu1—Cu2—I1^i^	−1.22 (4)	C17—P3—C25—C26	−160.4 (10)
Cu1^ii^—Cu1—Cu2—I1^i^	33.97 (4)	C21—P3—C25—C26	−59.1 (14)
Cu1^i^—Cu1—Cu2—I1^i^	−36.40 (4)	C17A—P3—C25—C26	169.6 (11)
P2—Cu1—Cu2—I1	−70.94 (13)	Cu3—P3—C25—C26	60.5 (10)
I1^ii^—Cu1—Cu2—I1	−150.28 (3)	P3—C25—C26—C27	−169.4 (10)
I2—Cu1—Cu2—I1	103.76 (4)	C25—C26—C27—C28	−165.3 (16)
Cu1^ii^—Cu1—Cu2—I1	138.94 (4)	C25—C26—C27—C28A	−92.2 (17)
Cu1^i^—Cu1—Cu2—I1	68.57 (4)	I4^iii^—Cu4—P4—C29^iv^	29.3 (5)
P2—Cu1—Cu2—I1^ii^	79.34 (13)	I4—Cu4—P4—C29^iv^	−90.7 (5)
I1—Cu1—Cu2—I1^ii^	150.28 (3)	I4^iv^—Cu4—P4—C29^iv^	149.3 (5)
I2—Cu1—Cu2—I1^ii^	−105.96 (4)	Cu3^iii^—Cu4—P4—C29^iv^	−30.6 (5)
Cu1^ii^—Cu1—Cu2—I1^ii^	−70.78 (4)	Cu3^iv^—Cu4—P4—C29^iv^	89.4 (5)
Cu1^i^—Cu1—Cu2—I1^ii^	−141.14 (4)	Cu3—Cu4—P4—C29^iv^	−150.6 (5)
P2—Cu1—Cu2—Cu1^ii^	150.12 (15)	I4^iii^—Cu4—P4—C29	−90.7 (5)
I1^ii^—Cu1—Cu2—Cu1^ii^	70.78 (4)	I4—Cu4—P4—C29	149.3 (5)
I1—Cu1—Cu2—Cu1^ii^	−138.94 (4)	I4^iv^—Cu4—P4—C29	29.3 (5)
I2—Cu1—Cu2—Cu1^ii^	−35.184 (9)	Cu3^iii^—Cu4—P4—C29	−150.6 (5)
Cu1^i^—Cu1—Cu2—Cu1^ii^	−70.369 (17)	Cu3^iv^—Cu4—P4—C29	−30.6 (5)
P2—Cu1—Cu2—Cu1^i^	−139.51 (15)	Cu3—Cu4—P4—C29	89.4 (5)
I1^ii^—Cu1—Cu2—Cu1^i^	141.14 (4)	I4^iii^—Cu4—P4—C29^iii^	149.3 (5)
I1—Cu1—Cu2—Cu1^i^	−68.57 (4)	I4—Cu4—P4—C29^iii^	29.3 (5)
I2—Cu1—Cu2—Cu1^i^	35.185 (9)	I4^iv^—Cu4—P4—C29^iii^	−90.7 (5)
Cu1^ii^—Cu1—Cu2—Cu1^i^	70.369 (17)	Cu3^iii^—Cu4—P4—C29^iii^	89.4 (5)
I1^i^—Cu2—P1—C1	101.6 (3)	Cu3^iv^—Cu4—P4—C29^iii^	−150.6 (5)
I1—Cu2—P1—C1	−18.4 (3)	Cu3—Cu4—P4—C29^iii^	−30.6 (5)
I1^ii^—Cu2—P1—C1	−138.4 (3)	C29^iv^—P4—C29—C30	−72.3 (11)
Cu1^ii^—Cu2—P1—C1	160.4 (3)	C29^iii^—P4—C29—C30	−179.1 (8)
Cu1^i^—Cu2—P1—C1	40.4 (3)	Cu4—P4—C29—C30	54.3 (9)
Cu1—Cu2—P1—C1	−79.6 (3)	P4—C29—C30—C31	178.9 (9)
I1^i^—Cu2—P1—C1^i^	−18.4 (3)	C29—C30—C31—C32	−177.6 (12)
I1—Cu2—P1—C1^i^	−138.4 (3)	Cu5^vi^—I6—Cu5—P6	144.84 (10)
I1^ii^—Cu2—P1—C1^i^	101.6 (3)	Cu6—I6—Cu5—P6	−144.27 (10)
Cu1^ii^—Cu2—P1—C1^i^	40.4 (3)	Cu5^vi^—I6—Cu5—I6^v^	−98.53 (5)
Cu1^i^—Cu2—P1—C1^i^	−79.6 (3)	Cu6—I6—Cu5—I6^v^	−27.64 (4)
Cu1—Cu2—P1—C1^i^	160.4 (3)	Cu5^vi^—I6—Cu5—I5	27.86 (5)
I1^i^—Cu2—P1—C1^ii^	−138.4 (3)	Cu6—I6—Cu5—I5	98.75 (5)
I1—Cu2—P1—C1^ii^	101.6 (3)	Cu5^vi^—I6—Cu5—Cu6	−70.89 (4)
I1^ii^—Cu2—P1—C1^ii^	−18.4 (3)	Cu6—I6—Cu5—Cu5^vi^	70.89 (4)
Cu1^ii^—Cu2—P1—C1^ii^	−79.6 (3)	Cu5^vi^—I6—Cu5—Cu5^v^	−35.39 (2)
Cu1^i^—Cu2—P1—C1^ii^	160.4 (3)	Cu6—I6—Cu5—Cu5^v^	35.50 (4)
Cu1—Cu2—P1—C1^ii^	40.4 (3)	Cu5^v^—I5—Cu5—P6	143.73 (12)
C1^i^—P1—C1—C2	66.6 (9)	Cu5^vi^—I5—Cu5—P6	−145.04 (12)
C1^ii^—P1—C1—C2	172.8 (6)	Cu5^v^—I5—Cu5—I6^v^	27.81 (5)
Cu2—P1—C1—C2	−60.3 (7)	Cu5^vi^—I5—Cu5—I6^v^	99.05 (4)
P1—C1—C2—C3	171.1 (6)	Cu5^v^—I5—Cu5—I6	−98.99 (4)
C1—C2—C3—C4	175.1 (8)	Cu5^vi^—I5—Cu5—I6	−27.75 (5)
I1^ii^—Cu1—P2—C13	94.7 (3)	Cu5^v^—I5—Cu5—Cu6	−35.619 (10)
I1—Cu1—P2—C13	−26.7 (3)	Cu5^vi^—I5—Cu5—Cu6	35.619 (10)
I2—Cu1—P2—C13	−144.2 (3)	Cu5^v^—I5—Cu5—Cu5^vi^	−71.238 (19)
Cu1^ii^—Cu1—P2—C13	159.4 (3)	Cu5^vi^—I5—Cu5—Cu5^v^	71.238 (19)
Cu1^i^—Cu1—P2—C13	−85.0 (3)	Cu5^vi^—I6—Cu6—P5	−143.97 (3)
Cu2—Cu1—P2—C13	30.7 (4)	Cu5—I6—Cu6—P5	143.95 (3)
I1^ii^—Cu1—P2—C5	−147.2 (3)	Cu5^vi^—I6—Cu6—I6^vi^	−27.51 (5)
I1—Cu1—P2—C5	91.4 (3)	Cu5—I6—Cu6—I6^vi^	−99.59 (7)
I2—Cu1—P2—C5	−26.0 (3)	Cu5^vi^—I6—Cu6—I6^v^	99.57 (7)
Cu1^ii^—Cu1—P2—C5	−82.4 (4)	Cu5—I6—Cu6—I6^v^	27.49 (5)
Cu1^i^—Cu1—P2—C5	33.1 (4)	Cu5^vi^—I6—Cu6—Cu5	72.08 (6)
Cu2—Cu1—P2—C5	148.8 (3)	Cu5^vi^—I6—Cu6—Cu5^v^	35.98 (5)
I1^ii^—Cu1—P2—C9	−25.4 (4)	Cu5—I6—Cu6—Cu5^v^	−36.09 (5)
I1—Cu1—P2—C9	−146.8 (4)	Cu5—I6—Cu6—Cu5^vi^	−72.08 (6)
I2—Cu1—P2—C9	95.8 (4)	P6—Cu5—Cu6—P5	1.1 (2)
Cu1^ii^—Cu1—P2—C9	39.4 (4)	I6^v^—Cu5—Cu6—P5	75.00 (5)
Cu1^i^—Cu1—P2—C9	154.9 (3)	I6—Cu5—Cu6—P5	−75.12 (5)
Cu2—Cu1—P2—C9	−89.4 (4)	I5—Cu5—Cu6—P5	180.0
C13—P2—C5—C6	67.1 (8)	Cu5^vi^—Cu5—Cu6—P5	−144.596 (12)
C9—P2—C5—C6	172.5 (7)	Cu5^v^—Cu5—Cu6—P5	144.597 (12)
Cu1—P2—C5—C6	−57.7 (7)	P6—Cu5—Cu6—I6	76.21 (17)
P2—C5—C6—C7	161.1 (8)	I6^v^—Cu5—Cu6—I6	150.11 (4)
C5—C6—C7—C8	56.1 (14)	I5—Cu5—Cu6—I6	−104.88 (5)
C13—P2—C9—C10	173.1 (8)	Cu5^vi^—Cu5—Cu6—I6	−69.48 (6)
C5—P2—C9—C10	67.0 (9)	Cu5^v^—Cu5—Cu6—I6	−140.29 (5)
Cu1—P2—C9—C10	−60.3 (8)	P6—Cu5—Cu6—I6^vi^	−178.84 (17)
P2—C9—C10—C11	−177.4 (8)	I6^v^—Cu5—Cu6—I6^vi^	−104.93 (2)
C9—C10—C11—C12	65.7 (14)	I6—Cu5—Cu6—I6^vi^	104.95 (2)
C5—P2—C13—C14	174.2 (8)	I5—Cu5—Cu6—I6^vi^	0.07 (5)
C9—P2—C13—C14	67.6 (8)	Cu5^vi^—Cu5—Cu6—I6^vi^	35.47 (6)
Cu1—P2—C13—C14	−61.3 (8)	Cu5^v^—Cu5—Cu6—I6^vi^	−35.33 (5)
P2—C13—C14—C15	174.3 (9)	P6—Cu5—Cu6—I6^v^	−73.90 (17)
C13—C14—C15—C16	−70.2 (18)	I6—Cu5—Cu6—I6^v^	−150.11 (4)
Cu3^iii^—I4—Cu3—P3	−146.42 (11)	I5—Cu5—Cu6—I6^v^	105.00 (5)
Cu4—I4—Cu3—P3	142.92 (11)	Cu5^vi^—Cu5—Cu6—I6^v^	140.41 (5)
Cu3^iii^—I4—Cu3—I4^iv^	98.66 (5)	Cu5^v^—Cu5—Cu6—I6^v^	69.60 (5)
Cu4—I4—Cu3—I4^iv^	28.00 (4)	P6—Cu5—Cu6—Cu5^v^	−143.5 (2)
Cu3^iii^—I4—Cu3—I3	−27.96 (5)	I6^v^—Cu5—Cu6—Cu5^v^	−69.60 (5)
Cu4—I4—Cu3—I3	−98.62 (5)	I6—Cu5—Cu6—Cu5^v^	140.29 (5)
Cu3^iii^—I4—Cu3—Cu4	70.66 (4)	I5—Cu5—Cu6—Cu5^v^	35.403 (12)
Cu4—I4—Cu3—Cu3^iii^	−70.66 (4)	Cu5^vi^—Cu5—Cu6—Cu5^v^	70.81 (2)
Cu3^iii^—I4—Cu3—Cu3^iv^	35.35 (2)	P6—Cu5—Cu6—Cu5^vi^	145.7 (2)
Cu4—I4—Cu3—Cu3^iv^	−35.31 (4)	I6^v^—Cu5—Cu6—Cu5^vi^	−140.41 (5)
Cu3^iii^—I3—Cu3—P3	144.02 (13)	I6—Cu5—Cu6—Cu5^vi^	69.48 (6)
Cu3^iv^—I3—Cu3—P3	−144.71 (13)	I5—Cu5—Cu6—Cu5^vi^	−35.404 (12)
Cu3^iii^—I3—Cu3—I4	27.94 (5)	Cu5^v^—Cu5—Cu6—Cu5^vi^	−70.81 (2)
Cu3^iv^—I3—Cu3—I4	99.20 (4)	I6—Cu6—P5—C33^v^	−37.2 (4)
Cu3^iii^—I3—Cu3—I4^iv^	−99.18 (4)	I6^vi^—Cu6—P5—C33^v^	−157.2 (4)
Cu3^iv^—I3—Cu3—I4^iv^	−27.91 (5)	I6^v^—Cu6—P5—C33^v^	82.8 (4)
Cu3^iii^—I3—Cu3—Cu4	−35.632 (10)	Cu5—Cu6—P5—C33^v^	22.9 (4)
Cu3^iv^—I3—Cu3—Cu4	35.634 (10)	Cu5^v^—Cu6—P5—C33^v^	142.9 (4)
Cu3^iv^—I3—Cu3—Cu3^iii^	71.27 (2)	Cu5^vi^—Cu6—P5—C33^v^	−97.1 (4)
Cu3^iii^—I3—Cu3—Cu3^iv^	−71.27 (2)	I6—Cu6—P5—C33	82.8 (5)
Cu3—I4—Cu4—P4	−144.01 (3)	I6^vi^—Cu6—P5—C33	−37.2 (5)
Cu3^iii^—I4—Cu4—P4	144.00 (3)	I6^v^—Cu6—P5—C33	−157.2 (5)
Cu3—I4—Cu4—I4^iii^	99.89 (7)	Cu5—Cu6—P5—C33	142.9 (5)
Cu3^iii^—I4—Cu4—I4^iii^	27.89 (5)	Cu5^v^—Cu6—P5—C33	−97.1 (5)
Cu3—I4—Cu4—I4^iv^	−27.91 (5)	Cu5^vi^—Cu6—P5—C33	22.9 (5)
Cu3^iii^—I4—Cu4—I4^iv^	−99.90 (7)	I6—Cu6—P5—C33^vi^	−157.2 (4)
Cu3—I4—Cu4—Cu3^iii^	71.99 (6)	I6^vi^—Cu6—P5—C33^vi^	82.8 (4)
Cu3—I4—Cu4—Cu3^iv^	35.97 (5)	I6^v^—Cu6—P5—C33^vi^	−37.2 (4)
Cu3^iii^—I4—Cu4—Cu3^iv^	−36.02 (5)	Cu5—Cu6—P5—C33^vi^	−97.1 (4)
Cu3^iii^—I4—Cu4—Cu3	−71.99 (6)	Cu5^v^—Cu6—P5—C33^vi^	22.9 (4)
P3—Cu3—Cu4—P4	0.5 (2)	Cu5^vi^—Cu6—P5—C33^vi^	142.9 (4)
I4—Cu3—Cu4—P4	74.87 (5)	C33^v^—P5—C33—C34	77.5 (12)
I4^iv^—Cu3—Cu4—P4	−74.93 (5)	C33^vi^—P5—C33—C34	−176.4 (8)
I3—Cu3—Cu4—P4	180.000 (2)	Cu6—P5—C33—C34	−49.4 (10)
Cu3^iii^—Cu3—Cu4—P4	144.581 (12)	P5—C33—C34—C35	−176.0 (9)
Cu3^iv^—Cu3—Cu4—P4	−144.578 (12)	C33—C34—C35—C36	−177.2 (11)
P3—Cu3—Cu4—I4^iii^	−179.43 (18)	I6^v^—Cu5—P6—C45	21.6 (4)
I4—Cu3—Cu4—I4^iii^	−105.10 (2)	I6—Cu5—P6—C45	142.2 (4)
I4^iv^—Cu3—Cu4—I4^iii^	105.10 (2)	I5—Cu5—P6—C45	−97.8 (4)
I3—Cu3—Cu4—I4^iii^	0.03 (5)	Cu6—Cu5—P6—C45	81.1 (4)
Cu3^iii^—Cu3—Cu4—I4^iii^	−35.39 (5)	Cu5^vi^—Cu5—P6—C45	−157.8 (4)
Cu3^iv^—Cu3—Cu4—I4^iii^	35.45 (5)	Cu5^v^—Cu5—P6—C45	−37.4 (5)
P3—Cu3—Cu4—I4	−74.33 (18)	I6^v^—Cu5—P6—C41	139.7 (4)
I4^iv^—Cu3—Cu4—I4	−149.80 (4)	I6—Cu5—P6—C41	−99.7 (4)
I3—Cu3—Cu4—I4	105.13 (5)	I5—Cu5—P6—C41	20.3 (4)
Cu3^iii^—Cu3—Cu4—I4	69.71 (5)	Cu6—Cu5—P6—C41	−160.8 (4)
Cu3^iv^—Cu3—Cu4—I4	140.55 (5)	Cu5^vi^—Cu5—P6—C41	−39.6 (5)
P3—Cu3—Cu4—I4^iv^	75.47 (18)	Cu5^v^—Cu5—P6—C41	80.7 (4)
I4—Cu3—Cu4—I4^iv^	149.80 (4)	I6^v^—Cu5—P6—C37	−90.7 (8)
I3—Cu3—Cu4—I4^iv^	−105.07 (5)	I6—Cu5—P6—C37	29.9 (8)
Cu3^iii^—Cu3—Cu4—I4^iv^	−140.49 (5)	I5—Cu5—P6—C37	149.8 (8)
Cu3^iv^—Cu3—Cu4—I4^iv^	−69.65 (5)	Cu6—Cu5—P6—C37	−31.3 (8)
P3—Cu3—Cu4—Cu3^iii^	−144.0 (2)	Cu5^vi^—Cu5—P6—C37	89.9 (8)
I4—Cu3—Cu4—Cu3^iii^	−69.71 (5)	Cu5^v^—Cu5—P6—C37	−149.8 (8)
I4^iv^—Cu3—Cu4—Cu3^iii^	140.49 (5)	I6^v^—Cu5—P6—C37A	−111.7 (7)
I3—Cu3—Cu4—Cu3^iii^	35.419 (12)	I6—Cu5—P6—C37A	8.8 (7)
Cu3^iv^—Cu3—Cu4—Cu3^iii^	70.84 (2)	I5—Cu5—P6—C37A	128.8 (7)
P3—Cu3—Cu4—Cu3^iv^	145.1 (2)	Cu6—Cu5—P6—C37A	−52.3 (8)
I4—Cu3—Cu4—Cu3^iv^	−140.55 (5)	Cu5^vi^—Cu5—P6—C37A	68.9 (8)
I4^iv^—Cu3—Cu4—Cu3^iv^	69.65 (5)	Cu5^v^—Cu5—P6—C37A	−170.8 (7)
I3—Cu3—Cu4—Cu3^iv^	−35.422 (11)	C45—P6—C37—C38	61 (2)
Cu3^iii^—Cu3—Cu4—Cu3^iv^	−70.84 (2)	C41—P6—C37—C38	−48 (2)
I4—Cu3—P3—C21A	157.5 (17)	C37A—P6—C37—C38	−43 (2)
I4^iv^—Cu3—P3—C21A	−83.0 (17)	Cu5—P6—C37—C38	−177.2 (19)
I3—Cu3—P3—C21A	37.4 (17)	P6—C37—C38—C39	−176.6 (16)
Cu4—Cu3—P3—C21A	−143.2 (17)	C45—P6—C37A—C38A	−64.0 (19)
Cu3^iii^—Cu3—P3—C21A	100.5 (17)	C41—P6—C37A—C38A	−166.7 (18)
Cu3^iv^—Cu3—P3—C21A	−25.5 (17)	C37—P6—C37A—C38A	18 (2)
I4—Cu3—P3—C17	−102.9 (7)	Cu5—P6—C37A—C38A	73.0 (19)
I4^iv^—Cu3—P3—C17	16.6 (7)	P6—C37A—C38A—C39	173.9 (15)
I3—Cu3—P3—C17	137.0 (7)	C37A—C38A—C39—C40	−52 (3)
Cu4—Cu3—P3—C17	−43.6 (8)	C37A—C38A—C39—C38	−36.0 (18)
Cu3^iii^—Cu3—P3—C17	−159.9 (7)	C37—C38—C39—C40	−169.9 (19)
Cu3^iv^—Cu3—P3—C17	74.1 (7)	C37—C38—C39—C38A	21.7 (16)
I4—Cu3—P3—C25	23.1 (5)	C45—P6—C41—C42	−168.3 (9)
I4^iv^—Cu3—P3—C25	142.6 (4)	C37—P6—C41—C42	−60.0 (12)
I3—Cu3—P3—C25	−97.0 (4)	C37A—P6—C41—C42	−62.0 (12)
Cu4—Cu3—P3—C25	82.5 (5)	Cu5—P6—C41—C42	66.2 (10)
Cu3^iii^—Cu3—P3—C25	−33.9 (5)	P6—C41—C42—C43	−164.3 (9)
Cu3^iv^—Cu3—P3—C25	−159.9 (4)	C41—C42—C43—C44	−65.7 (15)
I4—Cu3—P3—C21	136.8 (7)	C41—P6—C45—C46	−62.1 (10)
I4^iv^—Cu3—P3—C21	−103.8 (7)	C37—P6—C45—C46	179.3 (11)
I3—Cu3—P3—C21	16.6 (7)	C37A—P6—C45—C46	−156.1 (10)
Cu4—Cu3—P3—C21	−163.9 (7)	Cu5—P6—C45—C46	62.7 (10)
Cu3^iii^—Cu3—P3—C21	79.7 (7)	P6—C45—C46—C47	−174.7 (9)
Cu3^iv^—Cu3—P3—C21	−46.3 (7)	C45—C46—C47—C48	178.7 (11)

Symmetry codes: (i) −*y*+2, *x*−*y*+1, *z*; (ii) −*x*+*y*+1, −*x*+2, *z*; (iii) −*x*+*y*+1, −*x*+1, *z*; (iv) −*y*+1, *x*−*y*, *z*; (v) −*y*+1, *x*−*y*+1, *z*; (vi) −*x*+*y*, −*x*+1, *z*.

## References

[bb1] Ainscough, E. W., Brodie, A. M., Burrell, A. K., Freeman, G. H., Jameson, G. B., Bowmaker, G. A., Hanna, J. V. & Healy, P. C. (2001). *J. Chem. Soc. Dalton Trans.* pp. 144–151.

[bb2] Alyea, E. C., Ferguson, G., Malito, J. & Ruhl, B. L. (1985). *Inorg. Chem.* **24**, 3719–3720.

[bb3] Baker, L.-J., Bowmaker, G. A., Hart, R. D., Harvey, P. J., Healy, P. C. & White, A. H. (1994). *Inorg. Chem.* **33**, 3925–3931.

[bb4] Barron, P. F., Dyason, J. C., Engelhardt, L. M., Healy, P. C. & White, A. H. (1984). *Inorg. Chem.* **23**, 3766–3769.

[bb5] Blake, A. J., Brooks, N. R., Champness, N. R., Crew, M., Deveson, A., Fenske, D., Gregory, D. H., Hanton, L. R., Hubberstey, P. & Schröder, M. (2001). *Chem. Commun.* pp. 1432–1433.

[bb6] Blessing, R. H. (1995). *Acta Cryst.* A**51**, 33–38. 10.1107/s0108767394005726 7702794

[bb7] Bowmaker, G. A., Boyd, S. E., Hanna, J. V., Hart, R. D., Healy, P. C., Skelton, B. W. & White, A. H. (2002). *J. Chem. Soc. Dalton Trans.* pp. 2722–2730.

[bb8] Bowmaker, G. A., Camp, D., Hart, R. D., Healy, P. C., Skelton, B. W. & White, A. H. (1992). *Aust. J. Chem.* **45**, 1155–1166.

[bb9] Bowmaker, G. A., Cotton, J. D., Healy, P. C., Kildea, J. D., Silong, S. B., Skelton, B. W. & White, A. H. (1989). *Inorg. Chem.* **28**, 1462–1466.

[bb10] Bowmaker, G. A., de Silva, E. N., Healy, P. C., Skelton, B. W. & White, A. H. (1999). *J. Chem. Soc. Dalton Trans.* pp. 901–908.

[bb11] Bowmaker, G. A., Hanna, J. V., Hart, R. D., Healy, P. C. & White, A. H. (1994). *Aust. J. Chem.* **47**, 25–45.

[bb12] Bruker (2008). *APEX2* and *SAINT* Bruker AXS Inc., Madison, Wisconsin, USA.

[bb13] Churchill, M. R., Davies, G., El-Sayed, M. A., Hutchinson, J. P. & Rupich, M. W. (1982). *Inorg. Chem.* **21**, 995–1001.

[bb14] Churchill, M. R., DeBoer, B. G. & Donovan, D. J. (1975). *Inorg. Chem.* **14**, 617–623.

[bb15] Churchill, M. R., DeBoer, B. G. & Mendak, S. J. (1975). *Inorg. Chem.* **14**, 2041–2047.

[bb16] Churchill, M. R. & Kalra, K. L. (1973). *J. Am. Chem. Soc.* **95**, 5772–5773.

[bb17] Churchill, M. R. & Kalra, K. L. (1974). *Inorg. Chem.* **13**, 1065–1071.

[bb18] Churchill, M. R. & Rotella, F. J. (1977). *Inorg. Chem.* **16**, 3267–3273.

[bb19] Churchill, M. R. & Rotella, J. F. (1979). *Inorg. Chem.* **18**, 166–171.

[bb20] Dyason, J. C., Engelhardt, L. M., Pakawatchai, C., Healy, P. C. & White, A. H. (1985). *Aust. J. Chem.* **38**, 1243–1250.

[bb21] Dyason, J. C., Healy, P. C., Engelhardt, L. M., Pakawatchai, C., Patrick, V. A., Raston, C. L. & White, A. J. (1985). *J. Chem. Soc. Dalton Trans.* pp. 831–838.

[bb22] Flack, H. D. (1983). *Acta Cryst.* A**39**, 876–881.

[bb23] Gill, J. T., Mayerle, J. J., Welcker, P. S., Lewis, D. F., Ucko, D. A., Barton, D. J., Stowens, D. & Lippard, S. J. (1976). *Inorg. Chem.* **15**, 1155–1168.

[bb24] Glaeser, G. & Polthier, K. (2010). *Bilder der Mathematik*, p. 2. Heidelberg: Spektrum Akademischer Verlag.

[bb25] Goel, R. G. & Beauchamp, A. L. (1983). *Inorg. Chem.* **22**, 395–400.

[bb26] Hadjikakou, S. K., Akrivos, P. D., Karagiannidis, P., Raptopoulou, E. & Terzis, A. (1993). *Inorg. Chim. Acta*, **210**, 27–31.

[bb27] Herberhold, M., Akkus, N. & Milius, W. (2003). *Z. Anorg. Allg. Chem.* **629**, 2458–2464.

[bb28] Hermann, H. L., Boche, G. & Schwerdtfeger, P. (2001). *Chem. Eur. J.* **7**, 5333–5342. 10.1002/1521-3765(20011217)7:24<5333::aid-chem5333>3.0.co;2-1 11822433

[bb29] Jansen, M. (1987). *Angew. Chem.* **99**, 1136–1149.

[bb30] Kim, T. H., Shin, Y. W., Jung, J. H., Kim, J. S. & Kim, J. (2008). *Angew. Chem.* **120**, 697–700.

[bb31] Krause, N. (2002). In *Modern Organocopper Chemistry* Weinheim: Wiley-VCH.

[bb32] Mann, F. G., Purdie, D. & Wells, A. F. (1936). *J. Chem. Soc.* pp. 1503–1513.

[bb33] Medina, I., Mague, J. T. & Fink, M. J. (2005). *Acta Cryst.* E**61**, m1550–m1552.

[bb34] Moers, F. G. & Op Het Veld, P. H. (1970). *J. Inorg. Nucl. Chem.* **32**, 3225–3228.

[bb35] Ramaprabhu, S., Amstutz, N., Lucken, E. A. C. & Bernardinelli, G. (1993). *J. Chem. Soc. Dalton Trans.* pp. 871–875.

[bb36] Ramaprabhu, S., Amstutz, N., Lucken, E. A. C. & Bernardinelli, G. (1998). *Z. Naturforsch. Teil A*, **53**, 625–629.

[bb37] Schramm, V. (1978). *Inorg. Chem.* **17**, 714–718.

[bb38] Schwerdtfeger, P., Krawczyk, R. P., Hammerl, A. & Brown, R. (2004). *Inorg. Chem.* **43**, 6707–6716. 10.1021/ic0492744 15476370

[bb39] Sheldrick, G. M. (2008). *Acta Cryst.* A**64**, 112–122. 10.1107/S0108767307043930 18156677

[bb40] Soloveichik, G. L., Eisenstein, O., Poulton, J. T., Streib, W. E., Huffman, J. C. & Caulton, K. G. (1992). *Inorg. Chem.* **31**, 3306–3312.

[bb41] Spek, A. L. (2009). *Acta Cryst.* D**65**, 148–155. 10.1107/S090744490804362X PMC2631630 19171970

[bb42] Wells, A. F. (1936). *Z. Kristallogr.* **94**, 447–460.

[bb43] Whitesides, G. M., Casey, C. P. & Krieger, J. K. (1971). *J. Am. Chem. Soc.* **93**, 1379–1389.

